# Risk factors associated with revision for prosthetic joint infection following knee replacement: an observational cohort study from England and Wales

**DOI:** 10.1016/S1473-3099(18)30755-2

**Published:** 2019-06

**Authors:** Erik Lenguerrand, Michael R Whitehouse, Andrew D Beswick, Setor K Kunutsor, Pedro Foguet, Martyn Porter, Ashley W Blom, National Joint Registry for England, Wales, Northern Ireland and the Isle of Man

**Affiliations:** a Musculoskeletal Research Unit, Translational Health Sciences, Bristol Medical School, University of Bristol, Bristol, UK; b National Institute for Health Research Bristol Biomedical Research Centre, University Hospitals Bristol NHS Foundation Trust and University of Bristol, Bristol, UK; c University Hospitals Coventry and Warwickshire NHS Trust, Coventry, UK; d Centre for Hip Surgery, Wrightington Hospital, Wigan, UK

## Abstract

**Background:**

Prosthetic joint infection is a devastating complication of knee replacement. The risk of developing a prosthetic joint infection is affected by patient, surgical, and health-care system factors. Existing evidence is limited by heterogeneity in populations studied, short follow-up, inadequate power, and does not differentiate early prosthetic joint infection, most likely related to the intervention, from late infection, more likely to occur due to haematogenous bacterial spread. We aimed to assess the overall and time-specific associations of these factors with the risk of revision due to prosthetic joint infection following primary knee replacement.

**Methods:**

In this cohort study, we analysed primary knee replacements done between 2003 and 2013 in England and Wales and the procedures subsequently revised for prosthetic joint infection between 2003 and 2014. Data were obtained from the National Joint Registry linked to the Hospital Episode Statistics data in England and the Patient Episode Database for Wales. Each primary replacement was followed for a minimum of 12 months until the end of the observation period (Dec 31, 2014) or until the date of revision for prosthetic joint infection, revision for another indication, or death (whichever occurred first). We analysed the data using Poisson and piecewise exponential multilevel models to assess the associations between patient, surgical, and health-care system factors and risk of revision for prosthetic joint infection.

**Findings:**

Of 679 010 primary knee replacements done between 2003 and 2013 in England and Wales, 3659 were subsequently revised for an indication of prosthetic joint infection between 2003 and 2014, after a median follow-up of 4·6 years (IQR 2·6–6·9). Male sex (rate ratio [RR] for male *vs* female patients 1·8 [95% CI 1·7–2·0]), younger age (RR for age ≥80 years *vs* <60 years 0·5 [0·4–0·6]), higher American Society of Anaesthesiologists [ASA] grade (RR for ASA grade 3–5 *vs* 1, 1·8 [1·6–2·1]), elevated body-mass index (BMI; RR for BMI ≥30 kg/m^2^*vs* <25 kg/m^2^ 1·5 [1·3–1·6]), chronic pulmonary disease (RR 1·2 [1·1–1·3]), diabetes (RR 1·4 [1·2–1·5]), liver disease (RR 2·2 [1·6–2·9]), connective tissue and rheumatic diseases (RR 1·5 [1·3–1·7]), peripheral vascular disease (RR 1·4 [1·1–1·7]), surgery for trauma (RR 1·9 [1·4–2·6]), previous septic arthritis (RR 4·9 [2·7–7·6]) or inflammatory arthropathy (RR 1·4 [1·2–1·7]), operation under general anaesthesia (RR 1·1 [1·0–1·2]), requirement for tibial bone graft (RR 2·0 [1·3–2·7]), use of posterior stabilised fixed bearing prostheses (RR for posterior stabilised fixed bearing prostheses *vs* unconstrained fixed bearing prostheses 1·4 [1·3–1·5]) or constrained condylar prostheses (3·5 [2·5–4·7]) were associated with a higher risk of revision for prosthetic joint infection. However, uncemented total, patellofemoral, or unicondylar knee replacement (RR for uncemented *vs* cemented total knee replacement 0·7 [95% CI 0·6–0·8], RR for patellofemoral *vs* cemented total knee replacement 0·3 [0·2–0·5], and RR for unicondylar *vs* cemented total knee replacement 0·5 [0·5–0·6]) were associated with lower risk of revision for prosthetic joint infection. Most of these factors had time-specific effects, depending on the time period post-surgery.

**Interpretation:**

We have identified several risk factors for revision for prosthetic joint infection following knee replacement. Some of these factors are modifiable, and the use of targeted interventions or strategies could lead to a reduced risk of revision for prosthetic joint infection. Non-modifiable factors and the time-specific nature of the effects we have observed will allow clinicians to appropriately counsel patients preoperatively and tailor follow-up regimens.

**Funding:**

National Institute for Health Research.

## Introduction

Knee replacement is one of the most common elective surgical procedures worldwide. The National Joint Registry for England, Wales, Northern Ireland and the Isle of Man recorded 102 777 knee replacements performed in 2017, and the secular trend continues to increase.^1^ Deep infection is a rare but devastating complication affecting approximately 4% of primary and 15% of revision knee replacements.^2^ The most common causative organisms remain coagulase-negative staphylococci and *Staphylococcus aureus*, which are usually sensitive to a range of antibiotics.^3^ Treatment of prosthetic joint infection is expensive and protracted, and both the infection and the treatment have profoundly negative effects on patients and their families.^4^, ^5^, ^6^, ^7^ Treatment options include antibiotic suppression, debridement and retention of implants, excisional arthroplasty, and one-stage or two-stage revision.^8^, ^9^ However, all of these treatment options are associated with substantial morbidity and a high risk of adverse outcomes. As knee replacements have become more common, the number of revision operations for infection between 2005 and 2013 in England and Wales has risen by more than threefold, with more than 1000 revision procedures due to prosthetic joint infection of the knee done annually since 2011.^10^

**Research in context**

**Evidence before this study**

Prosthetic joint infection, although a rare complication following total joint replacement, is associated with devastating consequences. Evidence suggests that the risk of developing prosthetic joint infection following total hip or knee replacement is likely to be affected by patient-related, surgery-related, and health-care system-related factors. However, since total hip and knee replacements are two different operations involving patients with differing risk profiles, whether these factors affect prosthetic joint infection rates differentially in these patient groups remains uncertain. In a meta-analysis of 66 studies comprising more than 500 000 total joint replacements and published by our group in 2016, patient-related factors such as male sex, high body-mass index (BMI), steroid use, diabetes, rheumatoid arthritis, congestive heart failure, depression, and smoking and alcohol intake were each found to be associated with an increased risk of prosthetic joint infection. In a single cohort prospective study published in September, 2018, and comprising more than 600 000 primary hip replacements, we confirmed previous findings and showed several additional patient factors (eg, younger age, chronic pulmonary disease, and liver disease) and surgical factors (eg, surgery type, lateral surgical approach, and non-ceramic bearing surfaces) to be associated with an increased risk of infection. We also demonstrated that these factors exhibit specific time effects following surgery. However, the evidence for total knee replacements is less robust. We searched MEDLINE, Embase, and Web of Science from the date of the last search of the 2016 review (Sept 1, 2015) up to August, 2018, for observational cohort studies and systematic reviews and meta-analyses reporting on associations of patient-related, surgery-related, or health-care system-related factors with risk of prosthetic joint infection following total knee replacement. We used search terms related to the exposures (eg, “risk factor”, “body mass index”, and “comorbidity”) with those related to prosthetic joint infection (eg, “peri-prosthetic joint infection” and “prosthetic joint infection”). Our search was not restricted by language. We identified several registry studies and a meta-analysis based on 16 studies, whose findings are consistent with previous work on the topic. Existing evidence for the role of patient-related, surgery-related, or health-care system-related factors on prosthetic joint infection risk following total knee replacement is limited by inadequate sample sizes, short follow-up durations, inadequate adjustment for confounders, substantial inter-study heterogeneity, and inability to account for time-specific effects during follow-up.

**Added value of this study**

Using a single observational cohort of 679 010 primary total knee replacements, this study evaluated the overall and time-specific associations of patient, surgical, and health-care system factors on the risk of revision for prosthetic joint infection. Over a median follow-up of 4·6 years, 3659 knees were revised for prosthetic joint infection. Patient factors such as male sex, younger age (<60 years), high BMI (≥25 kg/m^2^), chronic pulmonary disease, diabetes, liver disease, connective tissue or rheumatic disease, and peripheral vascular disease were each associated with an increased risk of revision for prosthetic joint infection. Surgical factors such as indications for the primary procedure, type of procedure, and implant fixation and constraint or bearing significantly affected the risk of revision for prosthetic joint infection. Patients who received general anaesthesia or a tibial bone graft had an increased risk of revision, whereas the risk was lower for those who received a spinal anaesthetic. On the role of health-care system characteristics, high-volume hospitals had an increased risk of revision for prosthetic joint infection and privately funded procedures carried a lower risk of revision than operations funded by the NHS. Factors such as male sex and younger age had a consistent effect during the entire postoperative period, whereas other factors (such as indications for the primary procedure, type of procedure, and implant fixation and constraint or bearing) exhibited time-specific effects on revision for prosthetic joint infection.

**Implications of all the available evidence**

With the ageing population and a projected increase in total knee replacements, the burden of prosthetic joint infection will rise proportionately. The development of a prosthetic joint infection following total knee replacement is influenced by several modifiable and non-modifiable factors, which also seem to exhibit time-specific effects. Awareness of these factors and their time-specific effects should assist clinicians in appropriate counselling of patients preoperatively, optimisation of patients before surgery, as well as enhanced monitoring of at-risk patients after surgery.

The risk of developing infection after any form of arthroplasty is affected by both modifiable and non-modifiable patient, surgical, and health-care system factors. A recent systematic review of patient risk factors for prosthetic joint infection in both hip and knee replacements identified male sex, smoking, increasing body-mass index (BMI), steroid use, previous joint surgery, and comorbidities such as diabetes, rheumatoid arthritis, and depression, as notable risk factors for infection.^11^ However, limitations of this study and other reviews include short follow-up, pooled estimates based on variably adjusted data, and evidence of substantial heterogeneity between studies.^11^, ^12^

In view of these limitations, there is a need for large-scale cohort studies with adequate power to provide evidence about the nature and magnitude of the associations of potential risk factors with prosthetic joint infection. We recently published one such study about infection following hip replacement, which highlighted the importance of disentangling the time-specific effects of factors associated with early onset of prosthetic joint infection that are likely to be the consequence of the primary intervention versus factors associated with later onset that are more likely to result from haematogenous spread.^9^, ^13^

Although they are often studied together, hip and knee osteoarthritis are in some regards very different diseases^14^ with varying responses to joint replacement.^15^ Orthopaedic surgeons often specialise in either hip or knee replacement, and surgical techniques and implants aim to address specific issues relating to joint structure and function. Furthermore, patient recovery,^16^ outcomes,^17^ and rates of complications including prosthetic joint infection^10^, ^18^ differ between hip and knee replacement.

In this study, we aimed to assess the overall and post-operative period-specific associations of patient, surgical, and health-care setting factors with the risk of revision due to prosthetic joint infection in prospectively collected observational data of primary knee replacements done in England and Wales. We also aimed to investigate whether these factors differed from those associated with revision for prosthetic joint infection after hip replacement.

## Methods

### Study design and data sources

In this observational cohort study, we report analyses of data for England and Wales from the National Joint Registry for England, Wales, Northern Ireland, and the Isle of Man between April 1, 2003, and December 31, 2014. Data collection for Northern Ireland and the Isle of Man could not be considered due to their low number of procedures and insufficient duration of follow-up.

National Joint Registry data were linked to Hospital Episode Statistics in England and the Patient Episode Database for Wales to obtain data about inpatient and day case admissions. Data from the Office for National Statistics were linked to obtain the date of death.

Patient consent was obtained for data collection and linkage by the National Joint Registry. According to the NHS Health Research Authority, separate consent and ethics approval were not required for this study.

### Procedures

We analysed primary knee replacements done between April 1, 2003, and Dec 31, 2013, and revision procedures for prosthetic joint infection that occurred after the primary replacement between April 1, 2003, and Dec 31, 2014. The reason for revision is recorded by clinicians at the time of the revision procedure and reflects a clinical judgement sufficient to lead the surgeon to do an invasive procedure tailored to treat prosthetic joint infection. The diagnosis and treatment strategy for prosthetic joint infection is at the discretion of the surgeon and treating unit and is reflective of contemporary practice during the study period, with raised inflammatory markers, joint-specific symptoms, sinuses, and positive microbiological cultures^19^ being common diagnostic features during that period.

Each primary replacement was followed for a minimum of 12 months until the end of the observation period (Dec 31, 2014) or until the date of revision for prosthetic joint infection, revision for another indication, or death (whichever occurred first). Revisions for prosthetic joint infection included debridement and implant retention with modular exchange, or a single-stage or two-stage revision procedure.^20^

Patient characteristics^21^ considered were age, sex, ethnicity, BMI, American Society of Anesthesiologists (ASA) grade, and comorbidities. Ethnicity and comorbidities were obtained from the Hospital Episode Statistics records. We used International Classification of Diseases, 10th revision codes to classify comorbidities for which patients had been admitted to hospital in the 5 years preceding their primary operation (appendix p 2).^22^

Surgical factors^21^ considered were indication for surgery, anaesthesia type, thromboprophylaxis regime, surgical approach, knee replacement type, fixation, degree of constraint, use of bone graft, and occurrence of intraoperative complications (appendix p 2).

Health-care system factors^21^ considered were hospital type, funding source (National Health Service [NHS] or private), country, operating surgeon grade, consultant involvement, and the volume of knee surgeries (categorised into quartiles) done by the operating surgeon and surgeon in charge of the procedure in the preceding 12 months.

### Statistical analysis

The associations between the risk factors and risk of revision for prosthetic joint infection were first investigated during the overall follow-up period. Poisson multilevel models accounting for clustering at the unit level (random intercept) were used. Clustering at the surgeon level was negligible and therefore not considered further.

Prosthetic joint infection management can vary according to the time elapsed since the primary procedure at the time of onset of infection. Early onset of infection (within 2 years of the primary procedure) is generally believed to result from the primary intervention. Later onset of infection (2 years or longer after the primary procedure) is more likely to be due to haematogenous spread.^9^ For patients with early postoperative or acute haematogenous infection and a short duration of symptoms, debridement, modular exchange, and implant retention rather than full revision is appropriate if the joint replacement is well fixed.^9^ The associations between risk factors and risk of revision were therefore re-investigated over several at-risk post-operative periods: 0–3 months, 3–6 months, 6–12 months, 12–24 months, and more than 24 months. Each participant's at-risk period (defined as the time elapsed between their primary procedure and the endpoint) was split according to the time spent in each of these periods and the revision for prosthetic joint infection status (revised for prosthetic joint infection *vs* not) was assigned to the relevant period. We used a piecewise exponential multilevel model with period-specific effects to assess these associations—ie, their rate ratios (RRs) and 95% CIs across these time periods.^23^, ^24^ Analyses were done by running MLwiN from Stata version 14.1 using Markov Chain Monte Carlo methods.^25^ To account for test multiplicity, adjusted p values were derived using Simes' false-discovery rate testing controlling procedure.^26^, ^27^ To be confident that 95% of the effects tested were not due to chance, evidence of association was only discussed for adjusted p values of up to 0·05.

The analyses were done on the overall sample for all exposures except for ethnicity and comorbidities, which were only investigated in the patients operated on in England with record of hospital admission in Hospital Episode Statistics, but not in the Patient Episode Database for Wales and no evidence of residency outside England. The regressions were adjusted for age, sex, ASA grade, and BMI. BMI is an important risk factor for prosthetic joint infection but has substantial missing data in the National Joint Registry (47%), partly because it was not included as a variable in the early data collection forms. A missing at random mechanism was assumed to account for observed factors associated with the propensity of BMI to be missing and avoid the use of a complete-case analysis, which would have produced biased estimates: mean time at risk in missing BMI group 5·9 years (SD 2·8) versus 4·1 years (2·3) in the complete BMI group; incidence of revision for prosthetic joint infection 0·58 (95% CI 0·55–0·62) versus 1·68 (1·62–1·75); uncemented total knee replacement 6·0% versus 4·1%; other type of total knee replacement 1·6% versus 0·8%, unicondylar procedure 7·9% versus 9·3%. This approach also allowed us to use the entire study sample and investigate the rare exposure, something precluded with a complete case approach. A multiple imputation strategy was used to impute BMI using a Gaussian normal regression imputation model with the above factors used as covariates as well as the log of the observed event or censoring time, knee replacement type, and revision for prosthetic joint infection status. Because of the computational time required by each multilevel piecewise model, five imputations were computed and no sensitivity analyses of our missing at random approach were done. Estimates were combined by Rubin's rules. Unadjusted and adjusted models without BMI are available on request. To avoid over-adjustment, models investigating the effect of comorbidities were not adjusted for ASA grade, a proxy indicator of comorbidity profile.

### Role of the funding source

The funder of the study had no role in study design, data collection, data analysis, data interpretation, or writing of the report. EL had full access to all the data in the study. AWB is the guarantor and had final responsibility for the decision to submit for publication.

## Results

**Figure 1:**
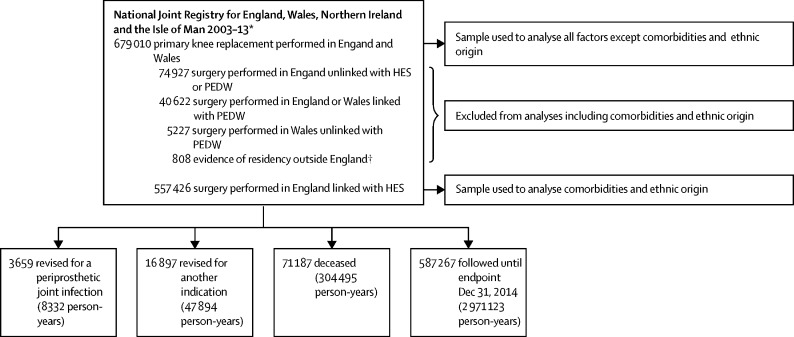
Study sample HES=Hospital Episode Statistics for England. PEDW=Patient Episode Database for Wales. *In this research, only data for England and Wales were considered; data collection for Northern Ireland commenced on Feb 1, 2013, and primary and revision procedures from this country could not be considered because of their low number and short follow-up. Data collection for the Isle of Man commenced on July 1, 2015, which was after the endpoint of the study and therefore these data were not considered. †As recorded in HES for the 5 years preceding the primary knee replacement.

Baseline study sample characteristics are presented in figure 1 and the table. 679 010 primary knee procedures were done in 449 different surgical units with a median of 1142 procedures (IQR 564–2144) per unit. Baseline characteristics were assessed in all 679 010 primary knee procedures, except for ethnicity and comorbidities, which were only investigated in the 557 426 patients operated on in England with record of hospital admission in Hospital Episode Statistics, but not in the Patient Episode Database for Wales and no evidence of residency outside England (figure 1, appendix p 2). 3659 index procedures were subsequently revised for an indication of prosthetic joint infection after a median follow-up of 4·6 years (IQR 2·6–6·9): 245 (6·7%) of these within 3 months, 238 (6·5%) between 3 and 6 months, 628 (17·2%) between 6 and 12 months, 970 (26·5%) between 1 and 2 years, and 1578 (43·1%) beyond 2 years from the index procedure. The median patient age was 70 years (IQR 63–76). The sample is presented by time periods in appendix pp 4–9. In 792 (28%) of the 2833 two-stage revision procedures done for prosthetic joint infection, only a second stage procedure was recorded in the National Joint Registry. Patients with incompletely registered two-stage procedures did not differ from those with complete procedures and their time to first stage procedure was estimated (appendix p 1).

**Table:** Sample description and incidence rates

		**n**	**Person-years of follow-up**	**Cases**	**Incidence rate per 1000 person-years (95% CI)**
**Patient characteristics**					
Sex					
	Female	386 047	1 913 858	1564	0·82 (0·78–0·86)
	Male	292 963	1 418 437	2095	1·48 (1·41–1·54)
Age, years					
	<60	109 000	537 080	793	1·48 (1·38–1·58)
	60–69	229 107	1 143 670	1342	1·17 (1·11–1·24)
	70–79	247 636	1 233 478	1223	0·99 (0·94–1·05)
	≥80	93 267	418 066	301	0·72 (0·64–0·81)
Ethnic origin					
	White	515 098	2 491 245	3066	1·23 (1·19–1·28)
	Black African origin	6011	27 217	52	1·91 (1·43–2·51)
	South Asian	15 510	69 500	94	1·35 (1·09–1·66)
	Other and mixed	6513	28 414	40	1·41 (1·01–1·92)
	Unclear	14 294	71 896	30	0·42 (0·28–0·60)
	Unavailable*	121 584	644 023	377	0·59 (0·53–0·65)
BMI, kg/m^2^					
	<25	40 333	167 416	212	1·27 (1·10–1·45)
	25–29·9	131 560	548 505	849	1·55 (1·45–1·66)
	≥30	205 134	840 041	1558	1·85 (1·76–1·95)
	Missing	301 983	1 776 333	1040	0·59 (0·55–0·62)
ASA grade					
	1	92 441	523 023	490	0·94 (0·86–1·02)
	2	484 992	2 347 559	2460	1·05 (1·01–1·09)
	3–5	101 577	461 713	709	1·54 (1·42–1·65)
Chronic pulmonary disease					
	No	478 788	2 350 416	2822	1·20 (1·16–1·25)
	Yes	78 638	337 856	460	1·36 (1·24–1·49)
	Unavailable*	121 584	644 023	377	0·59 (0·53–0·65)
Diabetes					
	No	490 521	2 398 439	2809	1·17 (1·13–1·22)
	Yes	66 905	289 833	473	1·63 (1·49–1·79)
	Unavailable*	121 584	644 023	377	0·59 (0·53–0·65)
Dementia					
	No	555 783	2 682 651	3274	1·22 (1·18–1·26)
	Yes	1643	5621	8	1·42 (0·61–2·80)
	Unavailable*	121 584	644 023	377	0·59 (0·53–0·65)
Liver disease					
	No	553 389	2 672 866	3237	1·21 (1·17–1·25)
	Yes	4037	15 406	45	2·92 (2·13–3·91)
	Unavailable*	121 584	644 023	377	0·59 (0·53–0·65)
Congestive heart failure					
	No	546 613	2 643 872	3211	1·21 (1·17–1·26)
	Yes	10 813	44 399	71	1·60 (1·25–2·02)
	Unavailable*	121 584	644 023	377	0·59 (0·53–0·65)
Connective tissue–rheumatic disease					
	No	526 493	2 547 415	3059	1·20 (1·16–1·24)
	Yes	30 933	140 856	223	1·58 (1·38–1·81)
	Unavailable*	121 584	644 023	377	0·59 (0·53–0·65)
Cancer					
	No	534 534	2 590 793	3170	1·22 (1·18–1·27)
	Non-metastatic cancer	20 364	87 849	95	1·08 (0·87–1·32)
	Metastatic cancer	2528	9629	17	1·77 (1·03–2·83)
	Unavailable*	121 584	644 023	377	0·59 (0·53–0·65)
Cerebrovascular disease					
	No	546 047	2 640 626	3221	1·22 (1·18–1·26)
	Yes	11 379	47 646	61	1·28 (0·98–1·64)
	Unavailable*	121 584	644 023	377	0·59 (0·53–0·65)
Myocardial infarction					
	No	541 849	2 619 164	3186	1·22 (1·17–1·26)
	Yes	15 577	69 107	96	1·39 (1·13–1·70)
	Unavailable*	121 584	644 023	377	0·59 (0·53–0·65)
Paraplegia and hemiplegia					
	No	555 229	2 678 669	3263	1·22 (1·18–1·26)
	Yes	2197	9603	19	1·98 (1·19–3·09)
	Unavailable*	121 584	644 023	377	0·59 (0·53–0·65)
Peptic ulcer disease					
	No	549 071	2 649 116	3229	1·22 (1·18–1·26)
	Yes	8355	39 155	53	1·35 (1·01–1·77)
	Unavailable*	121 584	644 023	377	0·59 (0·53–0·65)
Peripheral vascular disease					
	No	547 096	2 645 530	3207	1·21 (1·17–1·25)
	Yes	10 330	42 742	75	1·75 (1·38–2·20)
	Unavailable*	121 584	644 023	377	0·59 (0·53–0·65)
Renal disease					
	No	539 605	2 628 852	3197	1·22 (1·17–1·26)
	Yes	17 821	59 419	85	1·43 (1·14–1·77)
	Unavailable*	121 584	644 023	377	0·59 (0·53–0·65)
**Surgical characteristics**					
Osteoarthritis					
	No	18 529	92 371	157	1·70 (1·44–1·99)
	Yes	660 481	3 239 923	3502	1·08 (1·05–1·12)
Trauma					
	No	675 193	3 313 911	3613	1·09 (1·05–1·13)
	Yes	3817	18 383	46	2·50 (1·83–3·34)
Previous knee infection					
	No	678 522	3 329 986	3644	1·09 (1·06–1·13)
	Yes	488	2309	15	6·50 (3·64–10·72)
Avascular necrosis					
	No	676 515	3 319 900	3638	1·10 (1·06–1·13)
	Yes	2495	12 394	21	1·69 (1·05–2·59)
Inflammatory arthropathy					
	No	663 410	3 251 205	3534	1·09 (1·05–1·12)
	Yes	15 600	81 089	125	1·54 (1·28–1·84)
Other indication					
	No	675 312	3 317 114	3636	1·10 (1·06–1·13)
	Yes	3698	15 181	23	1·52 (0·96–2·27)
Surgical approach					
	Medial parapatellar	629 891	3 093 847	3 420	1·11 (1·07–1·14)
	Midvastus	19 384	89 860	76	0·85 (0·67–1·06)
	Lateral parapatellar	7992	42 506	47	1·11 (0·81–1·47)
	Subvastus	9403	48 926	49	1·00 (0·74–1·32)
	Other approach	12 340	57 155	67	1·17 (0·91–1·49)
Procedure					
	Primary TKR cemented	569 737	2 760 945	3227	1·17 (1·13–1·21)
	Primary TKR uncemented	33 754	188 639	153	0·81 (0·69–0·95)
	Primary TKR other	7699	48 490	52	1·07 (0·80–1·41)
	Unicondylar	58 885	291 906	211	0·72 (0·63–0·83)
	Patellofemoral	8935	42 314	16	0·38 (0·22–0·61)
Constraint					
	Unconstrained fixed	397 175	1 918 707	1987	1·04 (0·99–1·08)
	Unconstrained mobile	47 532	262 875	278	1·06 (0·94–1·19)
	Posterior stabilised fixed	144 960	705 782	981	1·39 (1·30–1·48)
	Posterior stabilised mobile	9714	51 054	54	1·06 (0·79–1·38)
	Constrained condylar	2968	11 498	43	3·74 (2·71–5·04)
	Fixed	16 703	74 967	67	0·89 (0·69–1·14)
	Mobile	41 297	212 086	141	0·66 (0·56–0·78)
	Undetermined	18 661	95 326	108	1·13 (0·93–1·37)
General anaesthesia					
	No	381 156	1 788 490	1889	1·06 (1·01–1·10)
	Yes	297 854	1 543 804	1770	1·15 (1·09–1·20)
Nerve block anaesthesia					
	No	547 783	2 644 393	2914	1·10 (1·06–1·14)
	Yes	131 227	687 902	745	1·08 (1·01–1·16)
Epidural anaesthesia					
	No	621 572	2 987 043	3311	1·11 (1·07–1·15)
	Yes	57 438	345 251	348	1·01 (0·90–1·12)
Spinal anaesthesia					
	No	283 120	1 499 422	1724	1·15 (1·10–1·21)
	Yes	395 890	1 832 873	1935	1·06 (1·01–1·10)
Thromboprophylaxis regimen					
	Chemical	606 001	2 870 437	3204	1·12 (1·08–1·16)
	Non-chemical	73 009	461 857	455	0·99 (0·90–1·08)
Femoral bone graft					
	No	675 147	3 317 925	3635	1·10 (1·06–1·13)
	Yes	3863	14 370	24	1·67 (1·07–2·49)
Tibial bone graft					
	No	676 271	3 319 182	3629	1·09 (1·06–1·13)
	Yes	2739	13 112	30	2·29 (1·54–3·27)
Intraoperative event					
	No	675 089	3 314 501	3636	..
	Yes	3921	17 794	23	1·29 (0·82–1·94)
**Health system characteristics**					
Country of surgery					
	England	638 835	3 136 010	3461	1·10 (1·07–1·14)
	Wales	40 175	196 285	198	1·01 (0·87–1·16)
Funding					
	NHS	574 433	2 722 013	3091	1·14 (1·10–1·18)
	Private	75 507	395 514	362	0·92 (0·82–1·01)
	Unspecified	29 070	214 768	206	0·96 (0·83–1·10)
Grade of operating surgeon					
	Consultant	572 464	2 767 937	3032	1·10 (1·06–1·14)
	Other	106 546	564 357	627	1·11 (1·03–1·20)
Consultant involvement					
	Operating	572 464	2 767 937	3032	1·10 (1·06–1·14)
	Assisting	38 327	188 754	223	1·18 (1·03–1·35)
	Not involved	68 219	375 604	404	1·08 (0·97–1·19)
Total volume (operating surgeon) of knee replacements done in previous 12 months					
	≤25	173 288	988 694	1091	1·10 (1·04–1·17)
	>25–50	160 104	815 890	983	1·20 (1·13–1·28)
	>50–85	170 157	788 139	816	1·04 (0·97–1·11)
	>85	175 461	739 571	769	1·04 (0·97–1·12)
Total volume (surgeon in charge) of knee replacements done in previous 12 months					
	≤38	173 204	1 010 739	1113	1·10 (1·04–1·17)
	>38–70	174 209	872 816	986	1·13 (1·06–1·20)
	>70–110	162 179	730 804	791	1·08 (1·01–1·16)
	>110	169 418	717 936	769	1·07 (1·00–1·15)
Total volume (hospital) of knee replacements done in previous 12 months					
	≤150	167 930	1 008 852	984	0·98 (0·92–1·04)
	>150–285	174 288	863 114	973	1·13 (1·06–1·20)
	>285–440	169 780	743 102	893	1·20 (1·12–1·28)
	>440	167 012	717 227	809	1·13 (1·05–1·21)

ASA=American Society of Anesthesiologists. TKR=total knee replacement. NHS=National Health Service.

* Information about ethnicity and comorbidities is only available for the 557 426 patients operated on in England with a Hospital Episodes Statistics record, no record in the Patient Episode Database for Wales, and no evidence of residency outside England. See figure 1 and appendix p 2 for more details.

**Figure 2:**
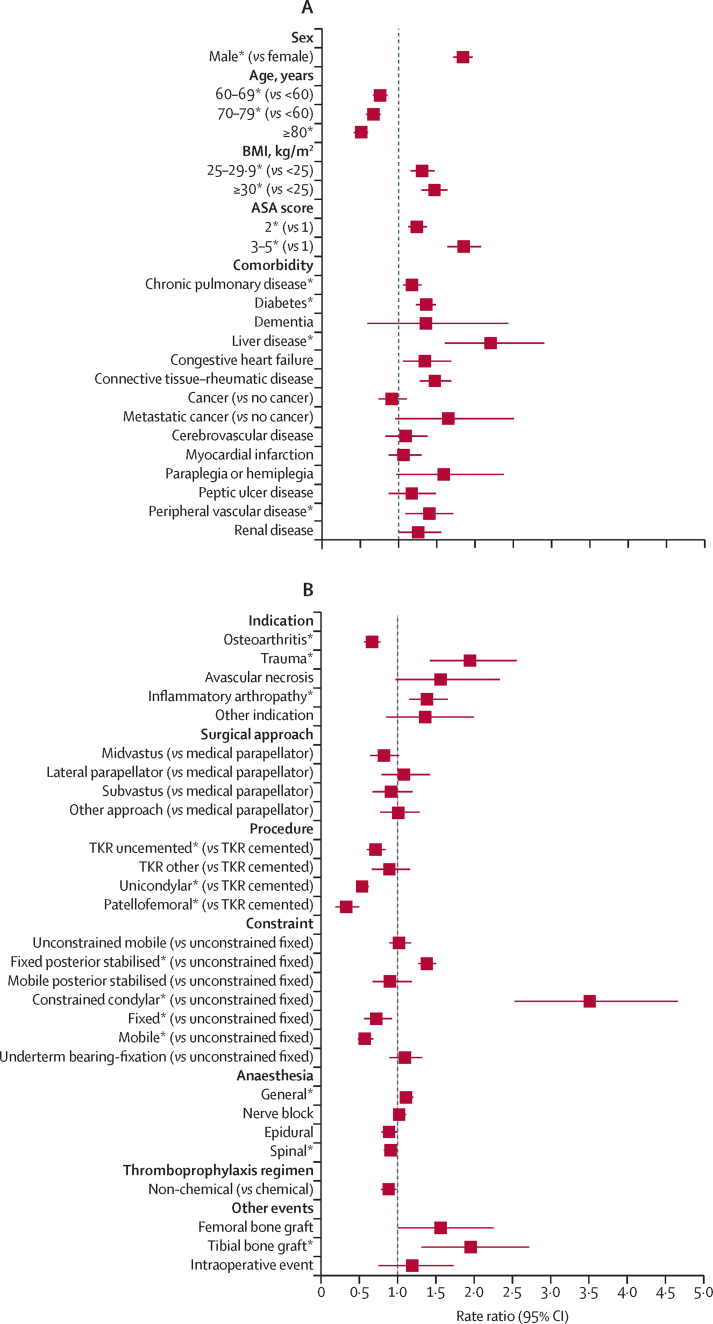

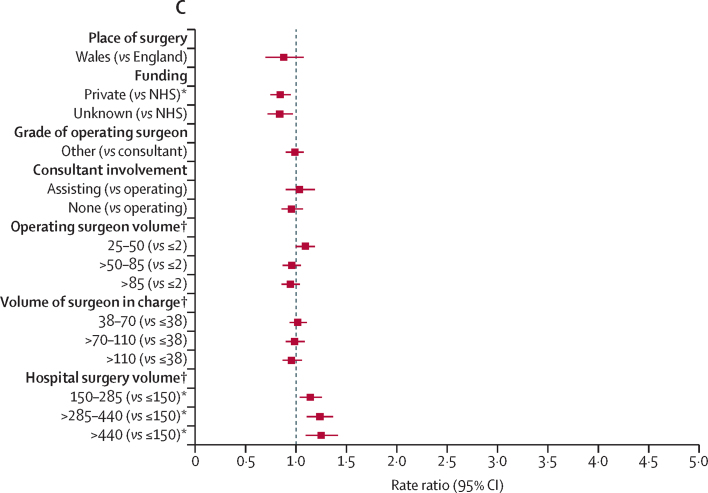
Risk factors of revision for prosthetic joint infection during the overall postoperative period (A) Patient factors. (B) Surgery factors. (C) Health-care system factors. Reference categories are in parentheses. BMI=body mass index. ASA=American Society of Anesthesiologists. TKR=total knee replacement. NHS=National Health Service. *Adjusted p value <0·05, detailed in appendix pp 10–12, alongside the rate ratios and 95% CIs. †Volume refers to the total volume of knee replacements done within the previous 12 months.

**Figure 3:**
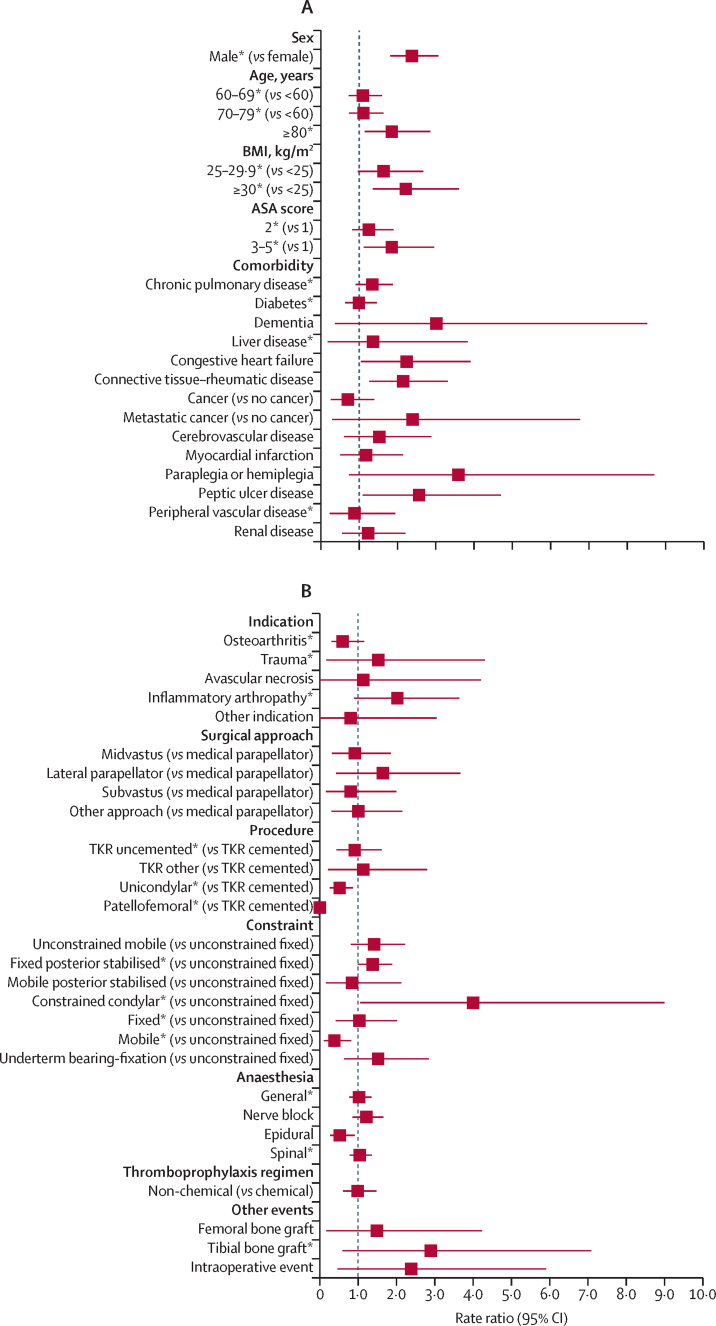

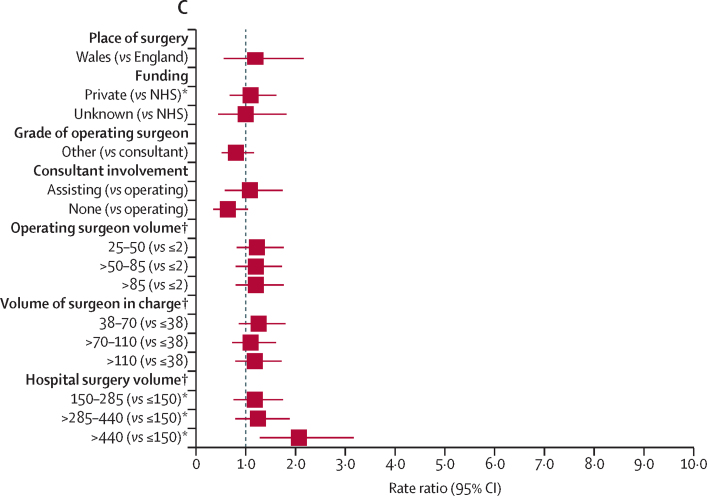
Risk factors of revision for prosthetic joint infection in the first 3 postoperative months (A) Patient factors. (B) Surgery factors. (C) Health-care system factors. Reference categories are in parentheses. BMI=body mass index. ASA=American Society of Anesthesiologists. TKR=total knee replacement. NHS=National Health Service. *Adjusted p value <0·05, detailed in appendix pp 10–12, alongside the rate ratios and 95% CIs. †Volume refers to the total volume of knee replacements performed within the previous 12 months.

RRs of revision for prosthetic joint infection surgery are presented in appendix pp 10–12. Figure 2 provides RRs over the entire follow-up and figure 3 shows their effect within the first 3 postoperative months. Effects associated with other periods are presented in appendix pp 15–18.

In terms of the role of patient characteristics, men were at a higher risk of revision for prosthetic joint infection in all time periods (figure 2). During the entire follow-up, the risk was lower for patients aged 60 years and older than in patients younger than 60 years of age. Patients aged 80 years and older were at increased risk of early revision for prosthetic joint infection but were at lower risk of revision thereafter (appendix pp 10, 15–18). Patients aged 60–79 years were at reduced risk of long-term revision for prosthetic joint infection (appendix pp 10, 15–18).

BMI of 30 kg/m^2^ or higher was associated with an increased risk of revision for prosthetic joint infection compared with BMI lower than 25 kg/m^2^ (figure 2), especially after the first year (appendix pp 10, 15–18). Compared with healthy patients, those with an ASA of 2 or higher were at increased risk (figure 2), especially beyond 6 months for ASA 3–5 and after 2 years for ASA 2 (appendix pp 10, 16–18).

Patients with a pre-existing history of chronic pulmonary disease, diabetes, liver disease, connective tissue or rheumatic disease, or peripheral vascular disease had a higher risk than those without pre-existing histories of these diseases (figure 2). Patients with a history of rheumatic disease had a higher risk of revision at most postoperative periods (appendix p 10). Those with liver disease were at higher risk of long-term revision than those without liver disease (appendix pp 10, 15–18). No or inconsistent time-specific effects were observed for the other comorbidities.

In terms of surgical characteristics, risk of revision for prosthetic joint infection varied according to the indication for the primary procedure. Patients operated on for osteoarthritis were less likely to be revised for prosthetic joint infection (figure 2). Those operated on for trauma, history of previous infection in the operated joint (appendix p 11), or an inflammation arthropathy were at increased risk of revision for prosthetic joint infection (figure 2), especially from 2 years post-operation (figure 3; appendix pp 11, 15–17). The indication for surgery did not affect the risk of early revision for prosthetic joint infection (appendix p 11). Patients operated on for trauma or with a history of previous infection were at an increased risk of revision from 1 year onwards (appendix pp 11, 17–18).

The risk of revision varied according to the type of procedure, implant fixation and constraint or bearing, with more extensive and complex procedures associated with an increased risk. Compared with cemented total knee replacement, patients who had an uncemented total knee replacement, a patellofemoral replacement, or an unicondylar replacement were at lower risk of revision for prosthetic joint infection (figure 2). From 6 months onwards, those with a unicondylar procedure were at lower risk of revision for prosthetic joint infection; the reduced risk of revision was observed from 1 year and 2 years onwards respectively for patellofemoral and uncemented total knee replacement procedures (appendix pp 11, 16–18).

The risk of revision was increased for patients with a posterior stabilised fixed-bearing implant or a constrained condylar implant compare with those with an unconstrained (or cruciate-retaining) fixed-bearing implant (figure 2): from 6 postoperative months onwards with a posterior stabilised fixed implant and beyond 1 year post-surgery for a constrained condylar implant (appendix pp 11, 16–18). The risk of revision was lower for patients with fixed or mobile bearing implants, and this finding was particularly evident from 6 months onwards for mobile bearing implants (appendix pp 11, 16–18).

The risk of revision for prosthetic joint infection was higher for patients who had received a general anaesthetic or tibial bone graft, and lower for those who had received a spinal anaesthetic. Little or no difference in the risk of revision for prosthetic joint infection was found for other anaesthetic techniques, thromboprophylaxis regimen, use of femoral bone graft, occurrence of intraoperative complication, or surgical approach.

In terms of health-care system characteristics, the risk of revision for prosthetic joint infection did not differ between Wales and England (figure 2). Privately funded procedures had a lower risk of revision than procedures funded by the NHS (figure 2), especially beyond 2 years (appendix pp 11, 18).

Revision for prosthetic joint infection was not affected by the grade of the operating surgeon, the presence or absence of a consultant surgeon during surgery, or by the volume of all knee procedures done by the operating surgeon or the surgeon in charge (Figure 2, Figure 3, appendix pp 11, 15–18).

The overall risk of revision for prosthetic joint infection was higher in high-volume hospitals than in low-volume hospitals (figure 2). Compared with hospitals with a small volume of activity, the risk of revision was higher in the first 3 months after primary surgery in hospitals that had done more than 440 knee procedures in the year preceding the index surgery (figure 3). No specific difference in the RRs were found beyond this period or for units doing lower volumes of knee procedures (appendix pp 11, 15–18).

## Discussion

The revision burden for prosthetic joint infection after knee replacement is higher than that after hip replacement in England and Wales.^10^, ^18^ In our cohort of 679 010 knee replacements, 3659 (0·53%) underwent revision for prosthetic joint infection compared with 2707 out of 623 253 (0·43%) hip replacements studied during the same period.^13^ However, revision within the first 3 months is proportionately less common (6·7% of knee replacements had revision surgery for infection *vs* 13·8% of hip replacements).

At the patient level, male patients, younger patients, and those with high BMI or more severe systemic disease, indicated by their ASA grade, had higher risk of revision for prosthetic join infection; however older patients (aged ≥80 years) were at high risk of early revision for prosthetic joint infection. This finding might reflect a tendency to treat older patients non-operatively with suppressive antibiotics in the longer term. Comorbidities that increased the risk of revision for prosthetic joint infection included chronic pulmonary disease, diabetes, liver disease, connective tissue or rheumatic diseases, and peripheral vascular disease. Treatment of these comorbidities and elevated BMI can potentially be optimised before surgery. A targeted preoperative intervention for male patients with high BMI and specific comorbidities could be a particularly relevant approach. Long-term vigilance seems to be important in those with liver disease. Our patient-level findings are concordant with the results of our study of infection after hip replacement,^13^ another large study of knee replacement,^28^ and systematic reviews.^11^, ^12^ Thus, these results might be generalisable to a wide population of patients undergoing implant surgery of various types.

At the surgical level, some of our results are consistent with those in hip surgery, but others are not. In particular, different surgical approaches in knee replacement are not associated with revision for prosthetic joint infection, but the use of general anaesthetic is. In general, factors that are a surrogate marker for operative duration and complexity, such as general anaesthetic, the need for additional constraint, total rather than partial knee replacement, and the use of tibial bone grafts, are associated with increased risk of revision for prosthetic joint infection. Concordant with hip surgery and previous studies,^11^, ^12^, ^28^ patients undergoing joint replacement for trauma or inflammatory arthritis have an increased risk of revision for infection. This finding is unsurprising because inflammatory arthropathies such as rheumatoid arthritis and drugs used to treat these conditions, such as disease-modifying antirheumatic drugs are known to be immunosuppressive.^29^ The substantially higher risk of prosthetic joint infection in those with historical infection of the knee is a new finding, but again unsurprising, and might be due to quiescent bacteria or other immune conditions that predispose individuals to infection.

Factors at the health-care system level seem to be less important than patient or surgical characteristics, with no notable sustained associations across the time periods studied. As previously reported,^28^ higher volume centres seemed to have a higher overall risk of revision for prosthetic joint infection and in the early postoperative period, but this association was not seen in the time-specific analysis or when test multiplicity was accounted for, indicating that this effect is not significant and might reflect more rapid diagnosis and early management of prosthetic joint infection in these centres. Privately funded patients were associated with lower long-term risks than those whose treatment was funded by the NHS—a finding not mirrored in our hip study. This difference is likely to reflect residual confounding with variables not available in our analysis because of case selection. The funding source of the primary procedure might therefore be a proxy for socioeconomic status, a patient factor not directly measured in the National Joint Registry.

Smoking has previously been identified as a risk factor for prosthetic joint infection,^11^, ^30^ and, although we did not have information about this, the surrogate comorbidity of chronic pulmonary disease was associated with increased risk. Evidence of an association between alcohol intake and increased risk has been inconsistent.^31^, ^32^ We noted a higher risk in patients with liver disease, but this outcome might represent a number of pathologies, including alcohol-related liver disease, and non-alcoholic-related disease such as fatty liver disease, hepatitis, haemochromatosis, or primary biliary cirrhosis. Our study corroborates the previous findings of increased risk in patients with diabetes and rheumatoid arthritis.^11^

The current study has several strengths. To our knowledge, this is the largest and most comprehensive evaluation of patient-related, surgical-related, and healthcare-related factors and their associations with the risk of revision for prosthetic joint infection of the knee. We used a large-scale cohort design comprising of a larger number of participants (>600 000) than those of the most up-to-date reviews on the topic (n=375 895 and n=512 508 hip and knee replacements)^11^, ^12^ or individual articles.^33^ Other strengths include the long-term follow-up of the cohort (median 4·6 years) and advanced statistical analyses, which include the evaluation of the effects of these potential risk factors in time-specific periods, which is appropriate because we have demonstrated that risk is not constant over time. There are also several limitations to our study. Although prospectively collected, our data are observational, and we can only draw inferences about the nature and magnitude of the associations, but not establish causation. In the UK, no agreed national gold standards are available to orthopaedic surgeons for the diagnosis of prosthetic joint infection. As such, the reported indication for revision of prosthetic joint infection in the National Joint Registry might vary between units. The approach used to diagnose prosthetic joint infection is, however, reflective of contemporary practice, with raised inflammatory markers, joint-specific symptoms, sinuses, and positive microbiological cultures.^19^ The diagnosis of prosthetic joint infection reflects a clinical judgement sufficient to lead the surgeon to conduct a very severe and invasive procedure tailored to tackle the infection. We also acknowledge issues relating to under-reporting of revision for prosthetic joint infection, and thus potentially lower incidence estimates.^34^ Linkage of the National Joint Registry data to microbiology data could reduce a posteriori any misdiagnoses of prosthetic joint infection, but has been shown to be of limited generalisability, with only 11·8% linkage achievable.^35^ The associations that we have identified might vary with different causative pathogens, but unfortunately we do not have the data to explore this concept. Our findings should be considered as conservative estimates of the risk factors with the strongest effects. The investigation into the effects of comorbidities was limited to a subset of National Joint Registry patients linked to Hospital Episode Statistics. This subset had higher ASA grade and therefore higher rate of revision for prosthetic joint infection than those excluded from these investigations, but they did not differ in terms of age, sex, BMI, or surgical characteristics, suggesting little evidence of differential selection bias. All other factors were investigated in the entire sample.

We have done appropriate modelling to adjust for known relevant confounders, but the possibility of residual confounding does exist. We had no specific data about confounders such as smoking and alcohol consumption, but have surrogate markers for these such as chronic pulmonary disease and liver disease. BMI data were not collected in the early years of the registry, necessitating imputation of the missing data, as with a previous study on this dataset.^36^ The duration of surgery is not collected in the National Joint Registry but the surgical characteristics influencing revision for prosthetic joint infection show that this factor is likely to have an important role: knee replacement type, fixation, and constraint are all associated with the duration, and complexity of surgery. This has previously been shown.^28^ Competing risk due to revision for another cause or death, which in combination affected 13% of the index primary knee replacements in the dataset during the period of observation (figure 1), could not be accounted for in the modelling strategy. This was a pragmatic decision because we chose a strategy focusing on time-specific effects while accounting for the clustering nature of the data, to disentangle the effects associated with surgical factors (likely to be more substantial in the short-term to mid-term follow-up) from those associated with health risk behaviours (likely to be more influential in the mid-term to long-term follow-up period). This strategy was optimal because there was evidence of non-proportional hazard rates (figure 3, appendix pp 15–18). Finally, it was not possible to investigate any ethnic disparities in terms of revision for prosthetic joint infection because of the small number of ethnic minority patients who underwent revision for prosthetic joint infection.

Knee replacement is an effective intervention to address the symptoms arising from degenerative knee conditions such as osteoarthritis. Although successful, complications can occur and prosthetic joint infection is a devastating example. Strategies should therefore be adopted to reduce the risk of infection. Modifiable risk factors could be ameliorated with targeted interventions that could lead to a reduction in the incidence of prosthetic joint infection. When risk factors are not modifiable, they should form part of the information used to counsel and prepare patients for surgery and can form the basis of targeted follow-up and monitoring strategies. The time period-specific effects of the identified risk factors should also form an integral part of the preparation for and management of knee replacement surgery. Overall, the results of this large cohort study could help to better inform the practice and delivery of knee replacement surgery.

## Data sharing

Data are accessible via application to the National Joint Registry Research Sub-Committee.
