# Supplementary material for: Risk factors associated with revision for prosthetic joint infection following knee replacement: an observational cohort study from England and Wales
Source: Lancet Infect Dis. 2019 Jun;19(6):589–600. doi: 10.1016/S1473-3099(18)30755-2 (PMC6531378; doi:10.1016/S1473-3099(18)30755-2)
Supplement: Supplementary appendix [file mmc1.pdf]

THE LANCET Infectious Diseases

Supplementary webappendix

This webappendix formed part of the original submission and has been peer reviewed. We post it as supplied by the authors.

Supplement to: Lenguerrand E, Whitehouse MR, Beswick AD, et al, on behalf of the National Joint Registry for England, Wales, Northern Ireland and the Isle of Man. Risk factors associated with revision for prosthetic joint infection following knee replacement: an observational cohort study from England and Wales. *Lancet Infect Dis* 2019; published online April 17. http://dx.doi.org/10.1016/S1473-3099(18)30755-2.

Web extra materials

Appendix 1

Patients with incompletely registered two-stage procedures did not differ from those with complete information at the time of their index surgery in terms of age (≥80y: 8% vs 8%; ≤60y: 22% vs 21%), sex (female: 43% vs 41%), BMI (<25kg/m^2: 8% vs 8%;≥30kg/m^2: 61% vs 60%), American Society of Anaesthesiologists (ASA) Physical Status grade (>P2: 20% vs 19%), or type of surgery (cemented total knee replacement: 90% vs 86%; uncemented total knee replacement: 4% vs 5%; unicondylar: 4% vs 6%, patellofemoral: 0.4% vs 0.4%).

For patients with incompletely registered two-stage revision procedures, the date of the first revision operation and the period they had been at risk of revision for PJI were estimated. We initially derived the relative weight of time elapsed between the index and the first operation by year and type of index surgery using patients with complete information: 100 x (length of time$_{\text{index surgery- 1st operation}}$/length of time $_{\text{index surgery- 2nd operation}}$). We then applied these weights to the length of time between the index and the second procedure for those with incomplete information to obtain an estimated "index surgery–1st operation" duration.

Appendix 2

Data on comorbidity were derived from the HES records. HES hospital admissions were linked to the NJR using a common anonymised patient identifier provided to NJR end-user by Northgate. The presence of each of the following comorbidities was identified using the ICD-10 codes associated with each hospital admission which occurred in the five years preceding the date of the primary replacement recorded in the NJR. The PEDW records were similarly linked but the dates of hospital admission were either missing or incomplete in the data extract available to us for numerous records preventing the accurate derivation of the five-year comorbidity presence. The comorbidity profile of patients with records in PEDW and/or operated in Wales could not be established with accuracy.

The analysis of the effect of specific comorbidity on revision for PJI was therefore restricted to patients with a knee replacement performed in England with linked HES record(s), with no record in the PEDW and no evidence of residency outside England. In other words, patients with their knee replacement performed in England but with PEDW record(s), patients with their knee replacement performed in Wales with or without PEDW/HES record(s) and patients with evidence of residency outside England were excluded. Moreover to reduce a risk of misclassification bias, patients operated in England with unlinked HES or PEDW record(s) could not be included in these analyses, i.e. they were not accounted as healthy patients: It was impossible to disentangle those who were genuinely healthy (i.e. absence of HES and PEDW record=true absence of any hospitalisation) from those who had been admitted to the hospital for any reason but for whom linkage between their NJR and HES/PEDW records could not be established(either due to missing data in the HES/PEDW file or of absence of match with the linkage method used by Northgate). No major evidence of difference was identified between included (n=557,426, figure1) and excluded (n=679,010-557,426=121,584, figure1) patients: median age 69y vs 68y, Male=43% vs 44%, median BMI 30 vs 30. However, those included had higher ASA grade (healthy patient(ASA=1): 13% vs 19%; mild systemic disease (ASA=2): 72% vs 69%; severe systemic disease (ASA=3): 15% vs 11%). This is expected as patients with HES records have at least one comorbidity and as explained above, those without a HES record are for some of them “healthy” patients with no comorbidity which had required an hospital admission.

The comorbidities considered were derived from the Charlson comorbidity index using the following ICD-10 codes:

- Myocardial infarction: I21.x, I22.x, I25.2
- Congestive heart failure: I09.9, I11.0, I13.0, I13.2, I25.5, I42.0, I42.5–I42.9, I43.x, I50.x, P29.0
- Peripheral vascular disease: I70.x, I71.x, I73.1, I73.8, I73.9, I77.1, I79.0, I79.2, K55.1, K55.8, K55.9, Z95.8, Z95.9
- Cerebrovascular disease: G45.x, G46.x, H34.0, I60.x–I69.x
- Dementia: F00.x–F03.x, F05.1, G30.x, G31.1
- Chronic pulmonary disease: I27.8, I27.9, J40.x–J47.x, J60.x–J67.x, J68.4, J70.1, J70.3
- Connective tissue disease or rheumatic disease: M05.x, M06.x, M31.5, M32.x–M34.x, M35.1, M35.3, M36.0
- Peptic ulcer disease: K25.x–K28.x
- Liver disease(mild and moderate or severe): B18.x, K70.0–K70.3, K70.9, K71.3–K71.5, K71.7, K73.x, K74.x, K76.0, K76.2–K76.4, K76.8, K76.9, Z94.4 + I85.0, I85.9, I86.4, I98.2, K70.4, K71.1, K72.1, K72.9, K76.5, K76.6, K76.7
- Diabetes (with and without chronic complication): E10.0, E10.1, E10.6, E10.8, E10.9, E11.0, E11.1, E11.6, E11.8, E11.9, E12.0, E12.1, E12.6, E12.8, E12.9, E13.0, E13.1, E13.6, E13.8, E13.9, E14.0, E14.1, E14.6, E14.8, E14.9+ E10.2–E10.5, E10.7, E11.2–E11.5, E11.7, E12.2–E12.5, E12.7, E13.2– E13.5, E13.7, E14.2–E14.5, E14.7
- Hemiplegia or paraplegia: G04.1, G11.4, G80.1, G80.2, G81.x, G82.x, G83.0–G83.4, G83.9
- Renal disease: I12.0, I13.1, N03.2–N03.7, N05.2– N05.7, N18.x, N19.x, N25.0, Z49.0– Z49.2, Z94.0, Z99.2
- Cancer(any malignancy and metastatic solid tumour): C00.x–C26.x, C30.x–C34.x, C37.x– C41.x, C43.x, C45.x–C58.x, C60.x– C76.x, C81.x–C85.x, C88.x, C90.x–C97.x+ C77.x–C80.x
- AIDS/HIV: B20.x–B22.x, B24.x

HIV/AIDS as a comorbidity group was not considered further in this research as only 45 patients had been admitted for this indication with none revised for a PJI

Methods of fixation and constraint of the implant were identified from the surgical approach section in the NJR data collection form and confirmed by the surgical procedure as identified from the dedicated field in the NJR form and from the component list of implants recorded in the component label section. Thromboprophylaxis regime was = classified as chemical (use of any chemical agent) and non-chemical (any other strategy). An intra-operative event was defined as the presence of any type of event reported by the surgeon. The grade, presence and surgical volume of a surgeon was derived using the anonymised ID of each surgeon. The place of surgery was identified using the post code of the hospital.

Appendix Table 1: Sample description and incidence rates by period of revision for PJI from the index procedures*

		0-3 months Person-years	Cases	Incidence[1]	95%CI[2]	3-6 months Person-years	Cases	Incidence	95%CI
**Patient characteristics**									
**Sex**	Female	94,880	91	0.96	[0.77, 1.18]	94,604	91	0.96	[0.77, 1.18]
	Male	71,928	154	2.14	[1.82, 2.51]	71,630	147	2.05	[1.73, 2.41]
**Age**	<60	26,824	35	1.30	[0.91, 1.81]	26,767	52	1.94	[1.45, 2.55]
	[60-69]	56,359	80	1.42	[1.13, 1.77]	56,248	70	1.24	[0.97, 1.57]
	[70-79]	60,830	83	1.36	[1.09, 1.69]	60,618	87	1.44	[1.15, 1.77]
	≥80	22,795	47	2.06	[1.51, 2.74]	22,602	29	1.28	[0.86, 1.84]
**Ethnicity**	White	126,553	190	1.50	[1.30, 1.73]	126,126	192	1.52	[1.31, 1.75]
	Black Afr. origin	1,475	3	2.03	[0.42, 5.94]	1,472	4	2.72	[0.74, 6.96]
	South Asian	3,809	7	1.84	[0.74, 3.79]	3,798	10	2.63	[1.26, 4.84]
	Other and mixed	1,600	3	1.87	[0.39, 5.48]	1,597	2	1.25	[0.15, 4.53]
	Unclear	3,501	1	0.29	[0.01, 1.59]	3,483	2	0.57	[0.07, 2.07]
**BMI[3]**	<25	9,901	11	1.11	[0.55, 1.99]	9,856	15	1.52	[0.85, 2.51]
	[25-29.9]	32,333	55	1.70	[1.28, 2.21]	32,228	66	2.05	[1.58, 2.61]
	≥30	50,431	111	2.20	[1.81, 2.65]	50,293	89	1.77	[1.42, 2.18]
	Missing	74,144	68	0.92	[0.71, 1.16]	73,857	68	0.92	[0.71, 1.17]
**ASA grade[4]**	1	22,736	26	1.14	[0.75, 1.68]	22,682	28	1.23	[0.82, 1.78]
	2	119,193	164	1.38	[1.17, 1.60]	118,840	162	1.36	[1.16, 1.59]
	3-5	24,879	55	2.21	[1.67, 2.88]	24,712	48	1.94	[1.43, 2.58]
**Chronic Pulmonary Disease**	No	117,631	169	1.44	[1.23, 1.67]	117,251	168	1.43	[1.22, 1.67]
	Yes	19,308	35	1.81	[1.26, 2.52]	19,225	42	2.18	[1.57, 2.95]
**Diabetes**	No	120,527	178	1.48	[1.27, 1.71]	120,136	175	1.46	[1.25, 1.69]
	Yes	16,412	26	1.58	[1.03, 2.32]	16,340	35	2.14	[1.49, 2.98]
**Dementia**	No	136,539	202	1.48	[1.28, 1.70]	136,084	208	1.53	[1.33, 1.75]
	Yes	400	2	5.00	[0.61, 18.06]	392	2	5.11	[0.62, 18.45]
**Liver Disease**	No	135,951	202	1.49	[1.29, 1.71]	135,496	207	1.53	[1.33, 1.75]
	Yes	987	2	2.03	[0.25, 7.32]	980	3	3.06	[0.63, 8.95]
**Congestive Heart Failure**	No	134,325	194	1.44	[1.25, 1.66]	133,899	205	1.53	[1.33, 1.76]
	Yes	2,614	10	3.83	[1.83, 7.03]	2,576	5	1.94	[0.63, 4.53]
**Connective Tissue-Rheumatologic Disease**	No	129,349	185	1.43	[1.23, 1.65]	128,924	191	1.48	[1.28, 1.71]
	Yes	7,590	19	2.50	[1.51, 3.91]	7,552	19	2.52	[1.51, 3.93]
**Cancer**	No	131,325	196	1.49	[1.29, 1.72]	130,905	201	1.54	[1.33, 1.76]
	Cancer	4,996	6	1.20	[0.44, 2.61]	4,962	6	1.21	[0.44, 2.63]
	Metastatic	618	2	3.24	[0.39, 11.69]	609	3	4.93	[1.02, 14.40]
**Cerebrovascular Disease**	No	134,171	197	1.47	[1.27, 1.69]	133,734	203	1.52	[1.32, 1.74]
	Yes	2,768	7	2.53	[1.02, 5.21]	2,741	7	2.55	[1.03, 5.26]
**Myocardial Infarction**	No	133,151	196	1.47	[1.27, 1.69]	132,716	208	1.57	[1.36, 1.80]
	Yes	3,788	8	2.11	[0.91, 4.16]	3,759	2	0.53	[0.06, 1.92]
**Paraplegia and Hemiplegia**	No	136,402	201	1.47	[1.28, 1.69]	135,944	208	1.53	[1.33, 1.75]
	Yes	537	3	5.59	[1.15, 16.34]	532	2	3.76	[0.46, 13.59]
**Peptic Ulcer Disease**	No	134,893	196	1.45	[1.26, 1.67]	134,442	208	1.55	[1.34, 1.77]
	Yes	2,046	8	3.91	[1.69, 7.71]	2,034	2	0.98	[0.12, 3.55]
**Peripheral Vascular Disease**	No	134,416	200	1.49	[1.29, 1.71]	133,973	202	1.51	[1.31, 1.73]
	Yes	2,523	4	1.59	[0.43, 4.06]	2,503	8	3.20	[1.38, 6.30]
**Renal Disease**	No	132,600	195	1.47	[1.27, 1.69]	132,178	202	1.53	[1.32, 1.75]
	Yes	4,339	9	2.07	[0.95, 3.94]	4,297	8	1.86	[0.80, 3.67]
**Surgical characteristics**									
**Osteoarthritis**	No	4,547	10	2.20	[1.05, 4.04]	4,518	11	2.43	[1.22, 4.36]
	Yes	162,262	235	1.45	[1.27, 1.65]	161,717	227	1.40	[1.23, 1.60]
**Trauma**	No	165,874	243	1.46	[1.29, 1.66]	165,307	236	1.43	[1.25, 1.62]

	Yes	934	2	2.14	[0.26, 7.73]	927	2	2.16	[0.26, 7.79]
**Previous knee infection**	No	166,688	244	1.46	[1.29, 1.66]	166,116	236	1.42	[1.25, 1.61]
	Yes	120	1	8.33	[0.21, 46.41]	119	2	16.81	[2.04, 60.71]
**Avascular necrosis**	No	166,196	244	1.47	[1.29, 1.66]	165,625	236	1.42	[1.25, 1.62]
	Yes	613	1	1.63	[0.04, 9.09]	610	2	3.28	[0.40, 11.85]
**Inflammatory arthropathy**	No	162,980	236	1.45	[1.27, 1.65]	162,426	228	1.40	[1.23, 1.60]
	Yes	3,829	9	2.35	[1.07, 4.46]	3,808	10	2.63	[1.26, 4.83]
**Other indication**	No	165,900	244	1.47	[1.29, 1.67]	165,334	236	1.43	[1.25, 1.62]
	Yes	908	1	1.10	[0.03, 6.14]	901	2	2.22	[0.27, 8.02]
**Surgical approach**	Medial parapatellar	154,743	227	1.47	[1.28, 1.67]	154,213	214	1.39	[1.21, 1.59]
	Mid Vastus	4,764	6	1.26	[0.46, 2.74]	4,750	5	1.05	[0.34, 2.46]
	Lateral parapatellar	1,961	4	2.04	[0.56, 5.22]	1,949	11	5.65	[2.82, 10.10]
	Sub Vastus	2,309	3	1.30	[0.27, 3.80]	2,300	3	1.30	[0.27, 3.81]
	Other approach	3,031	5	1.65	[0.54, 3.85]	3,022	5	1.65	[0.54, 3.86]
**Procedure[5]**	Prim TKR cemented	139,945	218	1.56	[1.36, 1.78]	139,463	195	1.40	[1.21, 1.61]
	Prim TKR uncemented	8,291	11	1.33	[0.66, 2.37]	8,259	18	2.18	[1.29, 3.44]
	Prim TKR other	1,891	3	1.59	[0.33, 4.64]	1,885	1	0.53	[0.01, 2.96]
	Unicondylar	14,484	13	0.90	[0.48, 1.53]	14,437	23	1.59	[1.01, 2.39]
	Patellofemoral	2,197	0			2,190	1	0.46	[0.01, 2.54]
**Constraint**	Unconstrained Fixed	97,580	132	1.35	[1.13, 1.60]	97,267	123	1.26	[1.05, 1.51]
	Unconstrained Mobile	11,674	20	1.71	[1.05, 2.65]	11,634	19	1.63	[0.98, 2.55]
	Posterior Stabilised Fixed	35,603	65	1.83	[1.41, 2.33]	35,471	59	1.66	[1.27, 2.15]
	Posterior Stabilised Mobile	2,387	3	1.26	[0.26, 3.67]	2,379	3	1.26	[0.26, 3.68]
	Constrained Condylar	724	4	5.52	[1.50, 14.14]	717	3	4.18	[0.86, 12.22]
	Fixed	4,110	8	1.95	[0.84, 3.84]	4,098	7	1.71	[0.69, 3.52]
	Mobile	10,157	5	0.49	[0.16, 1.15]	10,122	15	1.48	[0.83, 2.44]
	Undetermined	4,574	8	1.75	[0.76, 3.45]	4,547	9	1.98	[0.91, 3.76]
**General Anaesthesia**	No	93,638	138	1.47	[1.24, 1.74]	93,316	123	1.32	[1.10, 1.57]
	Yes	73,170	107	1.46	[1.20, 1.77]	72,919	115	1.58	[1.30, 1.89]
**Nerve Block Anaesthesia**	No	134,571	193	1.43	[1.24, 1.65]	134,106	193	1.44	[1.24, 1.66]
	Yes	32,238	52	1.61	[1.20, 2.12]	32,129	45	1.40	[1.02, 1.87]
**Epidural Anaesthesia**	No	152,709	233	1.53	[1.34, 1.73]	152,190	217	1.43	[1.24, 1.63]
	Yes	14,100	12	0.85	[0.44, 1.49]	14,045	21	1.50	[0.93, 2.29]
**Spinal Anaesthesia**	No	69,543	97	1.39	[1.13, 1.70]	69,298	113	1.63	[1.34, 1.96]
	Yes	97,266	148	1.52	[1.29, 1.79]	96,936	125	1.29	[1.07, 1.54]
**Thromboprophylaxis regimen**	Chemical	148,887	218	1.46	[1.28, 1.67]	148,379	214	1.44	[1.26, 1.65]
	Non-chemical	17,922	27	1.51	[0.99, 2.19]	17,855	24	1.34	[0.86, 2.00]
**Femoral bonegraft**	No	165,859	243	1.47	[1.29, 1.66]	165,290	238	1.44	[1.26, 1.63]
	Yes	950	2	2.11	[0.26, 7.61]	945	0		
**Tibial bonegraft**	No	166,136	242	1.46	[1.28, 1.65]	165,567	237	1.43	[1.25, 1.63]
	Yes	672	3	4.46	[0.92, 13.04]	667	1	1.50	[0.04, 8.35]
**Intraoperative event**	No	165,848	242	1.46	[1.28, 1.66]	165,279	238	1.44	[1.26, 1.64]
	Yes	960	3	3.12	[0.64, 9.13]	956	0		
**Health system characteristics**									
**Place of surgery**	England	156,941	229	1.46	[1.28, 1.66]	156,406	227	1.45	[1.27, 1.65]
	Wales	9,868	16	1.62	[0.93, 2.63]	9,828	11	1.12	[0.56, 2.00]
**Funding**	NHS	141,117	203	1.44	[1.25, 1.65]	140,641	196	1.39	[1.21, 1.60]
	Private	18,558	32	1.72	[1.18, 2.43]	18,492	34	1.84	[1.27, 2.57]
	Unspecified	7,133	10	1.40	[0.67, 2.58]	7,102	8	1.13	[0.49, 2.22]
**Grade operating surgeon**	Consultant	140,636	212	1.51	[1.31, 1.72]	140,155	190	1.36	[1.17, 1.56]
	Other	26,172	33	1.26	[0.87, 1.77]	26,079	48	1.84	[1.36, 2.44]
**Consultant involvement**	Operating	140,636	212	1.51	[1.31, 1.72]	140,155	190	1.36	[1.17, 1.56]
	Assisting	9,414	16	1.70	[0.97, 2.76]	9,379	19	2.03	[1.22, 3.16]
	Not involved	16,758	17	1.01	[0.59, 1.62]	16,701	29	1.74	[1.16, 2.49]
**Total volume**	≤25	42,558	54	1.27	[0.95, 1.66]	42,399	62	1.46	[1.12, 1.87]

**Operating surgeon**	]25-50]	39,316	61	1.55	[1.19, 1.99]	39,168	67	1.71	[1.33, 2.17]
	]50-85]	41,807	62	1.48	[1.14, 1.90]	41,673	52	1.25	[0.93, 1.64]
	>85	43,127	68	1.58	[1.22, 2.00]	42,994	57	1.33	[1.00, 1.72]
**Total volume Surgeon in charge**	≤38	42,533	54	1.27	[0.95, 1.66]	42,370	62	1.46	[1.12, 1.88]
	]38-70]	42,792	69	1.61	[1.25, 2.04]	42,648	65	1.52	[1.18, 1.94]
	]70-110]	39,847	56	1.41	[1.06, 1.83]	39,711	57	1.44	[1.09, 1.86]
	>110	41,637	66	1.59	[1.23, 2.02]	41,507	54	1.30	[0.98, 1.70]
**Total volume Hospital**	≤150	41,248	46	1.12	[0.82, 1.49]	41,090	60	1.46	[1.11, 1.88]
	]150-285]	42,819	57	1.33	[1.01, 1.72]	42,674	61	1.43	[1.09, 1.84]
	]285-440]	41,704	61	1.46	[1.12, 1.88]	41,564	60	1.44	[1.10, 1.86]
	>440	41,037	81	1.97	[1.57, 2.45]	40,907	57	1.39	[1.06, 1.81]

(continues on next page)

		6-12 months				1-2 years				> 2 years			
		**Person-years**	**Cases**	**Incidence[1]**	**95%CI[2]**	**Person-years**	**Cases**	**Incidence**	**95%CI**	**Person-years**	**Cases**	**Incidence**	**95%CI**
**Patient characteristics**													
**Sex**	Female	193,444	238	1.23	[1.08, 1.40]	353,962	407	1.15	[1.04, 1.27]	1,176,966	737	0.63	[0.58, 0.67]
	Male	146,168	390	2.67	[2.41, 2.95]	265,874	563	2.12	[1.95, 2.30]	862,836	841	0.97	[0.91, 1.04]
**Age**	<60	54,735	126	2.30	[1.92, 2.74]	99,675	193	1.94	[1.67, 2.23]	329,079	387	1.18	[1.06, 1.30]
	[60-69]	115,136	235	2.04	[1.79, 2.32]	210,567	367	1.74	[1.57, 1.93]	705,360	590	0.84	[0.77, 0.91]
	[70-79]	123,820	207	1.67	[1.45, 1.92]	226,692	335	1.48	[1.32, 1.64]	761,517	511	0.67	[0.61, 0.73]
	≥80	45,921	60	1.31	[1.00, 1.68]	82,903	75	0.90	[0.71, 1.13]	243,846	90	0.37	[0.30, 0.45]
**Ethnicity**	White	257,636	530	2.06	[1.89, 2.24]	468,868	816	1.74	[1.62, 1.86]	1,512,063	1,338	0.88	[0.84, 0.93]
	Black Afr. origin	3,007	8	2.66	[1.15, 5.24]	5,430	15	2.76	[1.55, 4.56]	15,833	22	1.39	[0.87, 2.10]
	South Asian	7,771	16	2.06	[1.18, 3.34]	13,954	29	2.08	[1.39, 2.98]	40,166	32	0.80	[0.54, 1.12]
	Other and mixed	3,266	10	3.06	[1.47, 5.63]	5,835	11	1.89	[0.94, 3.37]	16,115	14	0.87	[0.47, 1.46]
	Unclear	7,117	6	0.84	[0.31, 1.83]	12,882	7	0.54	[0.22, 1.12]	44,912	14	0.31	[0.17, 0.52]
**BMI[3]**	<25	20,097	42	2.09	[1.51, 2.82]	35,784	54	1.51	[1.13, 1.97]	91,779	90	0.98	[0.79, 1.21]
	[25-29.9]	65,836	140	2.13	[1.79, 2.51]	117,325	230	1.96	[1.72, 2.23]	300,783	358	1.19	[1.07, 1.32]
	≥30	102,817	253	2.46	[2.17, 2.78]	182,678	416	2.28	[2.06, 2.51]	453,822	689	1.52	[1.41, 1.64]
	Missing	150,863	193	1.28	[1.11, 1.47]	284,050	270	0.95	[0.84, 1.07]	1,193,420	441	0.37	[0.34, 0.41]
**ASA grade[4]**	1	46,400	80	1.72	[1.37, 2.15]	86,396	125	1.45	[1.20, 1.72]	344,808	231	0.67	[0.59, 0.76]
	2	242,929	415	1.71	[1.55, 1.88]	443,071	647	1.46	[1.35, 1.58]	1,423,526	1,072	0.75	[0.71, 0.80]
	3-5	50,283	133	2.65	[2.21, 3.13]	90,370	198	2.19	[1.90, 2.52]	271,469	275	1.01	[0.90, 1.14]
**Chronic Pulmonary Disease**	No	239,556	490	2.05	[1.87, 2.23]	437,171	744	1.70	[1.58, 1.83]	1,438,806	1,251	0.87	[0.82, 0.92]
	Yes	39,242	80	2.04	[1.62, 2.54]	69,798	134	1.92	[1.61, 2.27]	190,284	169	0.89	[0.76, 1.03]
**Diabetes**	No	245,476	483	1.97	[1.80, 2.15]	447,554	721	1.61	[1.50, 1.73]	1,464,747	1,252	0.85	[0.81, 0.90]
	Yes	33,323	87	2.61	[2.09, 3.22]	59,415	157	2.64	[2.25, 3.09]	164,343	168	1.02	[0.87, 1.19]
**Dementia**	No	278,011	570	2.05	[1.89, 2.23]	505,634	875	1.73	[1.62, 1.85]	1,626,384	1,419	0.87	[0.83, 0.92]
	Yes	788	0			1,335	3	2.25	[0.46, 6.56]	2,706	1	0.37	[0.01, 2.06]
**Liver Disease**	No	276,811	561	2.03	[1.86, 2.20]	503,551	865	1.72	[1.61, 1.84]	1,621,056	1,402	0.86	[0.82, 0.91]
	Yes	1,987	9	4.53	[2.07, 8.60]	3,418	13	3.80	[2.03, 6.50]	8,033	18	2.24	[1.33, 3.54]
**Congestive Heart Failure**	No	273,603	555	2.03	[1.86, 2.20]	497,788	860	1.73	[1.61, 1.85]	1,604,257	1,397	0.87	[0.83, 0.92]
	Yes	5,195	15	2.89	[1.62, 4.76]	9,181	18	1.96	[1.16, 3.10]	24,832	23	0.93	[0.59, 1.39]
**Connective Tissue-Rheumatologic Disease**	No	263,404	536	2.03	[1.87, 2.21]	479,292	822	1.72	[1.60, 1.84]	1,546,446	1,325	0.86	[0.81, 0.90]
	Yes	15,394	34	2.21	[1.53, 3.09]	27,677	56	2.02	[1.53, 2.63]	82,643	95	1.15	[0.93, 1.41]
**Cancer**	No	267,512	549	2.05	[1.88, 2.23]	487,052	849	1.74	[1.63, 1.86]	1,574,000	1,375	0.87	[0.83, 0.92]
	Cancer	10,071	19	1.89	[1.14, 2.95]	17,855	25	1.40	[0.91, 2.07]	49,965	39	0.78	[0.56, 1.07]
	Metastatic	1,215	2	1.65	[0.20, 5.94]	2,062	4	1.94	[0.53, 4.97]	5,125	6	1.17	[0.43, 2.55]
**Cerebrovascular Disease**	No	273,236	556	2.03	[1.87, 2.21]	497,129	864	1.74	[1.62, 1.86]	1,602,355	1,401	0.87	[0.83, 0.92]
	Yes	5,562	14	2.52	[1.38, 4.22]	9,840	14	1.42	[0.78, 2.39]	26,734	19	0.71	[0.43, 1.11]
**Myocardial Infarction**	No	271,158	551	2.03	[1.87, 2.21]	493,696	845	1.71	[1.60, 1.83]	1,588,443	1,386	0.87	[0.83, 0.92]
	Yes	7,640	19	2.49	[1.50, 3.88]	13,273	33	2.49	[1.71, 3.49]	40,647	34	0.84	[0.58, 1.17]
**Paraplegia and Hemiplegia**	No	277,718	566	2.04	[1.87, 2.21]	505,031	875	1.73	[1.62, 1.85]	1,623,574	1,413	0.87	[0.83, 0.92]
	Yes	1,080	4	3.70	[1.01, 9.48]	1,938	3	1.55	[0.32, 4.52]	5,516	7	1.27	[0.51, 2.61]
**Peptic Ulcer Disease**	No	274,652	562	2.05	[1.88, 2.22]	499,491	860	1.72	[1.61, 1.84]	1,605,637	1,403	0.87	[0.83, 0.92]
	Yes	4,146	8	1.93	[0.83, 3.80]	7,478	18	2.41	[1.43, 3.80]	23,452	17	0.72	[0.42, 1.16]
**Peripheral Vascular Disease**	No	273,715	557	2.03	[1.87, 2.21]	498,001	853	1.71	[1.60, 1.83]	1,605,426	1,395	0.87	[0.82, 0.92]
	Yes	5,084	13	2.56	[1.36, 4.37]	8,968	25	2.79	[1.80, 4.12]	23,664	25	1.06	[0.68, 1.56]
**Renal Disease**	No	270,075	551	2.04	[1.87, 2.22]	492,382	848	1.72	[1.61, 1.84]	1,601,617	1,401	0.87	[0.83, 0.92]
	Yes	8,723	19	2.18	[1.31, 3.40]	14,587	30	2.06	[1.39, 2.94]	27,473	19	0.69	[0.42, 1.08]
**Surgical characteristics**													
**Osteoarthritis**	No	9,209	29	3.15	[2.11, 4.52]	16,806	35	2.08	[1.45, 2.90]	57,293	72	1.26	[0.98, 1.58]
	Yes	330,404	599	1.81	[1.67, 1.96]	603,031	935	1.55	[1.45, 1.65]	1,982,510	1,506	0.76	[0.72, 0.80]
**Trauma**	No	337,723	619	1.83	[1.69, 1.98]	616,451	957	1.55	[1.46, 1.65]	2,028,556	1,558	0.77	[0.73, 0.81]
	Yes	1,889	9	4.76	[2.18, 9.04]	3,386	13	3.84	[2.04, 6.57]	11,247	20	1.78	[1.09, 2.75]
**Previous knee**	No	339,371	627	1.85	[1.71, 2.00]	619,404	966	1.56	[1.46, 1.66]	2,038,407	1,571	0.77	[0.73, 0.81]

**infection**	Yes	242	1	4.14	[0.10, 23.07]	432	4	9.26	[2.52, 23.70]	1,396	7	5.02	[2.02, 10.33]
**Avascular necrosis**	No	338,372	621	1.84	[1.69, 1.99]	617,579	965	1.56	[1.47, 1.66]	2,032,128	1,572	0.77	[0.74, 0.81]
	Yes	1,240	7	5.64	[2.27, 11.63]	2,257	5	2.22	[0.72, 5.17]	7,675	6	0.78	[0.29, 1.70]
**Inflammatory**	No	331,843	611	1.84	[1.70, 1.99]	605,571	943	1.56	[1.46, 1.66]	1,988,385	1,516	0.76	[0.72, 0.80]
**arthropathy**	Yes	7,769	17	2.19	[1.27, 3.50]	14,266	27	1.89	[1.25, 2.75]	51,418	62	1.21	[0.92, 1.55]
**Other indication**	No	337,780	626	1.85	[1.71, 2.00]	616,555	964	1.56	[1.47, 1.67]	2,031,545	1,566	0.77	[0.73, 0.81]
	Yes	1,832	2	1.09	[0.13, 3.94]	3,281	6	1.83	[0.67, 3.98]	8,258	12	1.45	[0.75, 2.54]
**Surgical approach**	Medial parapatellar	315,065	589	1.87	[1.72, 2.03]	575,016	906	1.58	[1.47, 1.68]	1,894,810	1,484	0.78	[0.74, 0.82]
	Mid Vastus	9,718	8	0.82	[0.36, 1.62]	17,676	24	1.36	[0.87, 2.02]	52,952	33	0.62	[0.43, 0.88]
	Lateral parapatellar	3,962	9	2.27	[1.04, 4.31]	7,278	9	1.24	[0.57, 2.35]	27,357	14	0.51	[0.28, 0.86]
	Sub Vastus	4,698	11	2.34	[1.17, 4.19]	8,608	13	1.51	[0.80, 2.58]	31,011	19	0.61	[0.37, 0.96]
	Other approach	6,169	11	1.78	[0.89, 3.19]	11,259	18	1.60	[0.95, 2.53]	33,673	28	0.83	[0.55, 1.20]
**Procedure[5]**	Prim TKR cemented	285,001	565	1.98	[1.82, 2.15]	518,791	861	1.66	[1.55, 1.77]	1,677,744	1,388	0.83	[0.78, 0.87]
	Prim TKR uncemented	16,862	21	1.25	[0.77, 1.90]	31,812	45	1.41	[1.03, 1.89]	123,415	58	0.47	[0.36, 0.61]
	Prim TKR other	3,846	10	2.60	[1.25, 4.78]	7,306	15	2.05	[1.15, 3.39]	33,562	23	0.69	[0.43, 1.03]
	Unicondylar	29,437	28	0.95	[0.63, 1.37]	53,765	47	0.87	[0.64, 1.16]	179,783	100	0.56	[0.45, 0.68]
	Patellofemoral	4,466	4	0.90	[0.24, 2.29]	8,162	2	0.25	[0.03, 0.89]	25,299	9	0.36	[0.16, 0.68]
**Constraint**	Unconstrained Fixed	198,807	351	1.77	[1.59, 1.96]	361,390	539	1.49	[1.37, 1.62]	1,163,663	842	0.72	[0.68, 0.77]
	Unconstrained Mobile	23,772	41	1.72	[1.24, 2.34]	44,716	75	1.68	[1.32, 2.10]	171,079	123	0.72	[0.60, 0.86]
	Posterior Stabilised Fixed	72,455	177	2.44	[2.10, 2.83]	132,236	255	1.93	[1.70, 2.18]	430,018	425	0.99	[0.90, 1.09]
	Posterior Stabilised Mobile	4,869	9	1.85	[0.85, 3.51]	8,999	12	1.33	[0.69, 2.33]	32,421	27	0.83	[0.55, 1.21]
	Constrained Condylar	1,454	7	4.82	[1.94, 9.92]	2,433	13	5.34	[2.85, 9.14]	6,170	16	2.59	[1.48, 4.21]
	Fixed	8,367	12	1.43	[0.74, 2.51]	14,882	14	0.94	[0.51, 1.58]	43,510	26	0.60	[0.39, 0.88]
	Mobile	20,627	15	0.73	[0.41, 1.20]	38,056	32	0.84	[0.58, 1.19]	133,124	74	0.56	[0.44, 0.70]
	Undetermined	9,262	16	1.73	[0.99, 2.81]	17,124	30	1.75	[1.18, 2.50]	59,819	45	0.75	[0.55, 1.01]
**General**	No	190,641	337	1.77	[1.58, 1.97]	344,669	518	1.50	[1.38, 1.64]	1,066,226	773	0.72	[0.67, 0.78]
**Anaesthesia**	Yes	148,971	291	1.95	[1.74, 2.19]	275,168	452	1.64	[1.49, 1.80]	973,577	805	0.83	[0.77, 0.89]
**Nerve Block**	No	273,948	506	1.85	[1.69, 2.02]	496,637	776	1.56	[1.45, 1.68]	1,605,131	1,246	0.78	[0.73, 0.82]
**Anaesthesia**	Yes	65,664	122	1.86	[1.54, 2.22]	123,200	194	1.57	[1.36, 1.81]	434,672	332	0.76	[0.68, 0.85]
**Epidural**	No	310,936	585	1.88	[1.73, 2.04]	565,745	881	1.56	[1.46, 1.66]	1,805,464	1,395	0.77	[0.73, 0.81]
**Anaesthesia**	Yes	28,676	43	1.50	[1.09, 2.02]	54,092	89	1.65	[1.32, 2.02]	234,339	183	0.78	[0.67, 0.90]
**Spinal Anaesthesia**	No	141,553	280	1.98	[1.75, 2.22]	262,560	435	1.66	[1.50, 1.82]	956,468	799	0.84	[0.78, 0.90]
	Yes	198,059	348	1.76	[1.58, 1.95]	357,277	535	1.50	[1.37, 1.63]	1,083,335	779	0.72	[0.67, 0.77]
**Thromboprophylaxis**	Chemical	303,177	563	1.86	[1.71, 2.02]	550,163	869	1.58	[1.48, 1.69]	1,719,831	1,340	0.78	[0.74, 0.82]
**regimen**	Non-chemical	36,435	65	1.78	[1.38, 2.27]	69,673	101	1.45	[1.18, 1.76]	319,972	238	0.74	[0.65, 0.84]
**Femoral**	No	337,685	625	1.85	[1.71, 2.00]	616,486	961	1.56	[1.46, 1.66]	2,032,605	1,568	0.77	[0.73, 0.81]
**bonegraft**	Yes	1,927	3	1.56	[0.32, 4.55]	3,350	9	2.69	[1.23, 5.10]	7,198	10	1.39	[0.67, 2.56]
**Tibial**	No	338,250	624	1.84	[1.70, 2.00]	617,363	957	1.55	[1.45, 1.65]	2,031,865	1,569	0.77	[0.73, 0.81]
**bonegraft**	Yes	1,362	4	2.94	[0.80, 7.52]	2,473	13	5.26	[2.80, 8.99]	7,938	9	1.13	[0.52, 2.15]
**Intraoperative**	No	337,664	624	1.85	[1.71, 2.00]	616,265	962	1.56	[1.46, 1.66]	2,029,445	1,570	0.77	[0.74, 0.81]
**event**	Yes	1,949	4	2.05	[0.56, 5.26]	3,571	8	2.24	[0.97, 4.41]	10,358	8	0.77	[0.33, 1.52]
**Health system characteristics**													
**Place of surgery**	England	319,530	602	1.88	[1.74, 2.04]	582,849	920	1.58	[1.48, 1.68]	1,920,284	1,483	0.77	[0.73, 0.81]
	Wales	20,083	26	1.29	[0.85, 1.90]	36,987	50	1.35	[1.00, 1.78]	119,519	95	0.79	[0.64, 0.97]
**Funding**	NHS	287,368	527	1.83	[1.68, 2.00]	521,939	844	1.62	[1.51, 1.73]	1,630,947	1,321	0.81	[0.77, 0.85]
	Private	37,755	75	1.99	[1.56, 2.49]	69,767	81	1.16	[0.92, 1.44]	250,943	140	0.56	[0.47, 0.66]
	Unspecified	14,489	26	1.79	[1.17, 2.63]	28,130	45	1.60	[1.17, 2.14]	157,913	117	0.74	[0.61, 0.89]
**Grade operating**	Consultant	286,344	531	1.85	[1.70, 2.02]	521,237	794	1.52	[1.42, 1.63]	1,679,564	1,305	0.78	[0.74, 0.82]
**surgeon**	Other	53,268	97	1.82	[1.48, 2.22]	98,599	176	1.79	[1.53, 2.07]	360,239	273	0.76	[0.67, 0.85]
**Consultant**	Operating	286,344	531	1.85	[1.70, 2.02]	521,237	794	1.52	[1.42, 1.63]	1,679,564	1,305	0.78	[0.74, 0.82]
**involvement**	Assisting	19,152	32	1.67	[1.14, 2.36]	34,909	61	1.75	[1.34, 2.24]	115,900	95	0.82	[0.66, 1.00]
	Not involved	34,116	65	1.91	[1.47, 2.43]	63,690	115	1.81	[1.49, 2.17]	244,338	178	0.73	[0.63, 0.84]
**Total volume**	≤25	86,598	171	1.97	[1.69, 2.29]	161,050	287	1.78	[1.58, 2.00]	656,089	517	0.79	[0.72, 0.86]
**Operating surgeon**	]25-50]	79,993	128	1.60	[1.33, 1.90]	146,990	264	1.80	[1.59, 2.03]	510,422	463	0.91	[0.83, 0.99]
	]50-85]	85,162	166	1.95	[1.66, 2.27]	154,650	215	1.39	[1.21, 1.59]	464,846	321	0.69	[0.62, 0.77]

	>85	87,858	163	1.86	[1.58, 2.16]	157,145	204	1.30	[1.13, 1.49]	408,446	277	0.68	[0.60, 0.76]
**Total volume Surgeon in charge**	≤38	86,541	165	1.91	[1.63, 2.22]	161,902	287	1.77	[1.57, 1.99]	677,393	545	0.80	[0.74, 0.88]
	]38-70]	87,111	150	1.72	[1.46, 2.02]	159,352	243	1.52	[1.34, 1.73]	540,914	459	0.85	[0.77, 0.93]
	]70-110]	81,140	154	1.90	[1.61, 2.22]	146,942	231	1.57	[1.38, 1.79]	423,165	293	0.69	[0.62, 0.78]
	>110	84,820	159	1.87	[1.59, 2.19]	151,641	209	1.38	[1.20, 1.58]	398,331	281	0.71	[0.63, 0.79]
**Total volume Hospital**	≤150	83,910	155	1.85	[1.57, 2.16]	157,634	222	1.41	[1.23, 1.61]	684,970	501	0.73	[0.67, 0.80]
	]150-285]	87,194	155	1.78	[1.51, 2.08]	159,839	268	1.68	[1.48, 1.89]	530,588	432	0.81	[0.74, 0.89]
	]285-440]	84,916	160	1.88	[1.60, 2.20]	151,101	261	1.73	[1.52, 1.95]	423,818	351	0.83	[0.74, 0.92]
	>440	83,592	158	1.89	[1.61, 2.21]	151,263	219	1.45	[1.26, 1.65]	400,427	294	0.73	[0.65, 0.82]

1. Incidence rate per 1,000 person-years
2. 95%CI: 95% confidence interval
3. BMI: Body Mass Index (kg/m^2)
4. ASA: American Society of Anaesthesiologists grade.
5. TKR: Total Knee Replacement

*Information on ethnicity and comorbidities are only available on the 557,426 patients operated in England with a HES record, no record in PEDW and no evidence of residency outside England. See figure 1 and Appendix 2 page 2 for more details.

Appendix Table 2: Incidence rate ratios of revision for PJI and 95% confidence intervals for patient characteristics

		Total				≤3mths				3–6mths				6–12mths				12–24mths				≥24mths			
		**RR**	**95%CI**	**P-value**	**Adj. p-value**	**RR**	**95%CI**	**P-value**	**Adj. p-value**	**RR**	**95%CI**	**P-value**	**Adj. p-value**	**RR**	**95%CI**	**P-value**	**Adj. p-value**	**RR**	**95%CI**	**P-value**	**Adj. p-value**	**RR**	**95%CI**	**P-value**	**Adj. p-value**
**Sex**	**Male**	1.83	[1.71-1.96]	<0.0001	<0.0001	2.37	[1.81-3.06]	<0.0001	<0.0001	2.17	[1.65-2.81]	<0.0001	<0.0001	2.19	[1.86-2.57]	<0.0001	<0.0001	1.85	[1.62-2.09]	<0.0001	<0.0001	1.57	[1.42-1.74]	<0.0001	<0.0001
	**Female**	Ref				Ref				Ref				Ref				Ref				Ref			
DIC		53,378				61,493																			
	**≥80**	0.50	[0.43-0.57]	<0.0001	<0.0001	1.85	[1.14-2.85]	0.01	0.05	0.64	[0.38-0.99]	0.05	0.16	0.57	[0.40-0.77]	<0.0001	<0.0001	0.48	[0.36-0.62]	<0.0001	<0.0001	0.32	[0.25-0.40]	<0.0001	<0.0001
**Age**	**[70–79]**	0.65	[0.60-0.72]	<0.0001	<0.0001	1.11	[0.73-1.63]	0.69	0.87	0.70	[0.49-0.98]	0.04	0.14	0.71	[0.56-0.88]	<0.0001	0.01	0.75	[0.62-0.89]	<0.0001	0.01	0.56	[0.49-0.64]	<0.0001	<0.0001
	**[60–69]**	0.76	[0.69-0.83]	<0.0001	<0.0001	1.09	[0.72-1.59]	0.76	0.90	0.61	[0.42-0.86]	0.01	0.03	0.85	[0.68-1.05]	0.13	0.31	0.87	[0.73-1.03]	0.10	0.26	0.69	[0.60-0.78]	<0.0001	<0.0001
	**<60**	Ref				Ref				Ref				Ref				Ref				Ref			
DIC		53,378				61,493																			
**Ethnicity**	**Black African origin**	1.67	[1.24-2.17]	<0.0001	<0.0001	1.70	[0.35-4.20]	0.58	0.79	2.05	[0.55-4.60]	0.28	0.54	1.47	[0.63-2.69]	0.38	0.63	1.79	[0.99-2.82]	0.06	0.17	1.64	[1.02-2.41]	0.03	0.12
	**Other and mixed**	1.17	[0.84-1.57]	0.36	0.61	1.43	[0.29-3.49]	0.78	0.91	0.88	[0.10-2.48]	0.62	0.81	1.59	[0.75-2.76]	0.21	0.44	1.16	[0.57-1.96]	0.74	0.89	0.98	[0.53-1.57]	0.84	0.93
	**South Asian**	1.17	[0.93-1.43]	0.17	0.38	1.51	[0.59-2.90]	0.41	0.65	1.89	[0.88-3.30]	0.09	0.23	1.14	[0.64-1.78]	0.72	0.88	1.33	[0.88-1.88]	0.18	0.39	0.91	[0.61-1.26]	0.54	0.75
	**Unclear**	0.35	[0.23-0.48]	<0.0001	<0.0001	0.20	[0.01-0.76]	0.02	0.07	0.39	[0.05-1.09]	0.13	0.31	0.39	[0.14-0.77]	0.02	0.07	0.31	[0.12-0.58]	<0.0001	0.01	0.35	[0.19-0.55]	<0.0001	<0.0001
	**White**	Ref				Ref				Ref				Ref				Ref				Ref			
DIC		46,938				54,314																			
	**≥30**	1.46	[1.29-1.63]	<0.0001	<0.0001	2.23	[1.35-3.60]	0.02	0.09	1.34	[0.83-2.10]	0.39	0.63	1.26	[0.96-1.64]	0.12	0.31	1.52	[1.19-1.92]	<0.0001	0.02	1.46	[1.22-1.73]	<0.0001	<0.0001
**BMI[1]**	**[25–29·9]**	1.30	[1.15-1.46]	<0.0001	<0.0001	1.64	[0.96-2.67]	0.25	0.49	1.54	[0.94-2.42]	0.19	0.41	1.11	[0.84-1.46]	0.53	0.75	1.39	[1.08-1.77]	0.02	0.07	1.27	[1.05-1.53]	0.02	0.09
	**<25**	Ref				Ref				Ref				Ref				Ref				Ref			
DIC		53,378				61,493																			
**ASA[2]**	**3–5**	1.84	[1.63-2.07]	<0.0001	<0.0001	1.85	[1.11-2.95]	0.02	0.08	1.89	[1.13-3.00]	0.02	0.07	1.80	[1.34-2.39]	<0.0001	<0.0001	1.71	[1.35-2.15]	<0.0001	<0.0001	1.76	[1.46-2.11]	<0.0001	<0.0001
	**2**	1.23	[1.12-1.36]	<0.0001	<0.0001	1.25	[0.81-1.89]	0.36	0.61	1.29	[0.85-1.93]	0.27	0.53	1.11	[0.87-1.41]	0.43	0.67	1.11	[0.91-1.34]	0.33	0.59	1.27	[1.10-1.47]	<0.0001	0.01
	**1**	Ref				Ref				Ref				Ref				Ref				Ref			
DIC		53,378				61,493																			
**Chronic Pulmonary Disease**		1.17	[1.05-1.29]	<0.0001	0.02	1.33	[0.90-1.87]	0.15	0.36	1.59	[1.10-2.19]	0.01	0.05	1.04	[0.81-1.31]	0.77	0.91	1.16	[0.96-1.39]	0.13	0.31	1.03	[0.87-1.20]	0.75	0.90
DIC		47,073				54,367																			
**Diabetes**		1.35	[1.22-1.48]	<0.0001	<0.0001	0.99	[0.63-1.45]	0.89	0.95	1.43	[0.96-2.01]	0.07	0.21	1.27	[0.99-1.58]	0.05	0.16	1.57	[1.31-1.86]	<0.0001	<0.0001	1.17	[0.99-1.37]	0.06	0.18
DIC		47,049				54,343																			
**Dementia**		1.35	[0.58-2.43]	0.52	0.74	3.02	[0.36-8.52]	0.30	0.56	3.39	[0.40-9.51]	0.25	0.49					1.51	[0.31-3.64]	0.72	0.88	0.54	[0.01-1.99]	0.35	0.61
DIC		47,083				54,370																			
**Liver disease**		2.20	[1.60-2.90]	<0.0001	<0.0001	1.36	[0.17-3.83]	0.97	0.98	1.89	[0.38-4.59]	0.48	0.70	2.09	[0.95-3.70]	0.06	0.17	2.05	[1.08-3.31]	0.02	0.08	2.32	[1.37-3.51]	<0.0001	0.01
DIC		47,062				54,351																			
**Congestive Heart Failure**		1.34	[1.05-1.68]	0.02	0.07	2.23	[1.04-3.90]	0.03	0.10	1.21	[0.38-2.51]	0.86	0.94	1.42	[0.79-2.25]	0.23	0.47	1.15	[0.68-1.75]	0.63	0.82	1.16	[0.73-1.69]	0.56	0.77
DIC		47,078				54,367																			
**Rheumatologic Disease**		1.47	[1.27-1.68]	<0.0001	<0.0001	2.15	[1.26-3.31]	<0.0001	0.02	1.95	[1.15-3.00]	0.01	0.04	1.27	[0.87-1.75]	0.22	0.44	1.33	[1.00-1.72]	0.05	0.15	1.40	[1.13-1.72]	<0.0001	0.01
DIC		47,056				54,348																			
**Cancer**	**Cancer**	0.90	[0.73-1.10]	0.30	0.56	0.70	[0.25-1.39]	0.31	0.56	0.76	[0.27-1.49]	0.39	0.63	0.90	[0.54-1.36]	0.58	0.79	0.80	[0.52-1.16]	0.25	0.49	0.96	[0.68-1.30]	0.76	0.91
	**Metastatic**	1.64	[0.95-2.50]	0.06	0.18	2.40	[0.29-6.77]	0.46	0.70	3.60	[0.73-8.74]	0.08	0.23	0.92	[0.11-2.55]	0.66	0.84	1.24	[0.34-2.72]	0.88	0.95	1.52	[0.56-2.95]	0.44	0.68
	**None**	Ref				Ref				Ref				Ref				Ref				Ref			
DIC		47,080				54,373																			
**Cerebrovascular Disease**		1.08	[0.82-1.37]	0.62	0.81	1.52	[0.60-2.88]	0.39	0.63	1.63	[0.64-3.08]	0.31	0.56	1.24	[0.68-1.98]	0.51	0.74	0.84	[0.45-1.33]	0.43	0.67	0.88	[0.53-1.33]	0.52	0.74
DIC		47,083				54,371																			
**Myocardial Infarction**		1.06	[0.86-1.29]	0.58	0.79	1.17	[0.50-2.14]	0.81	0.92	0.30	[0.04-0.83]	0.06	0.18	1.09	[0.65-1.65]	0.80	0.92	1.34	[0.91-1.85]	0.13	0.31	0.95	[0.65-1.30]	0.70	0.88
DIC		47,083				54,368																			
**Paraplegia and Hemiplegia**		1.59	[0.96-2.37]	0.06	0.18	3.59	[0.73-8.71]	0.08	0.23	2.35	[0.28-6.56]	0.47	0.70	1.78	[0.49-3.93]	0.40	0.65	0.88	[0.18-2.13]	0.63	0.82	1.45	[0.58-2.71]	0.45	0.69
DIC		47,080				54,373																			
**Peptic Ulcer Disease**		1.15	[0.86-1.48]	0.37	0.61	2.56	[1.09-4.70]	0.02	0.08	0.63	[0.08-1.78]	0.37	0.61	0.97	[0.42-1.76]	0.80	0.92	1.44	[0.85-2.18]	0.16	0.37	0.88	[0.51-1.35]	0.52	0.74
DIC		47,082				54,370																			
**Peripheral Vascular Disease**		1.37	[1.08-1.71]	0.01	0.04	0.87	[0.23-1.94]	0.62	0.81	1.91	[0.82-3.50]	0.12	0.29	1.15	[0.61-1.87]	0.72	0.88	1.53	[0.98-2.20]	0.06	0.16	1.22	[0.79-1.76]	0.38	0.63
DIC		47,077				54,366																			
**Renal Disease**		1.26	[1.00-1.55]	0.04	0.14	1.23	[0.55-2.20]	0.68	0.86	1.22	[0.52-2.24]	0.73	0.89	1.13	[0.67-1.71]	0.70	0.88	1.31	[0.87-1.84]	0.18	0.40	0.90	[0.54-1.36]	0.58	0.79
DIC		47,079				54,376																			

RR: Rate ratio adjusted for age, sex, Body Mass Index and ASA grade; CI: 95% Confidence Interval; Adj. p-value: q-value or adjusted p-value. DIC: Deviance Information Criterion (RRs and 95%CI for ≤3mths to ≥24mths periods are estimated from the same piece-wise exponential multilevel model)

1. Body Mass Index: kg/m2
2. American Society of Anaesthesiologists grade of surgical fitness.

Appendix Table 3: Incidence rate ratios of revision for PJI and 95% confidence intervals for surgical characteristics

		Total				≤3mths				3–6mths				6–12mths				12–24mths				≥24mths			
		RR	95%CI	P-value	Adj. p-value	RR	95%CI	P-value	Adj. p-value	RR	95%CI	P-value	Adj. p-value	RR	95%CI	P-value	Adj. p-value	RR	95%CI	P-value	Adj. p-value	RR	95%CI	P-value	Adj. p-value
**Osteoarthritis**		0.66	[0.56-0.77]	<0.0001	<0.0001	0.61	[0.32-1.17]	0.10	0.26	0.63	[0.33-1.17]	0.11	0.28	0.58	[0.39-0.84]	<0.0001	0.02	0.76	[0.54-1.07]	0.10	0.26	0.68	[0.53-0.87]	<0.0001	0.01
DIC		53,355				61,461																			
**Trauma**		1.94	[1.42-2.55]	<0.0001	<0.0001	1.53	[0.18-4.33]	0.85	0.93	1.26	[0.15-3.53]	0.96	0.98	2.23	[1.01-3.94]	0.03	0.12	2.16	[1.15-3.50]	0.01	0.05	1.86	[1.13-2.78]	0.01	0.04
DIC		53,363				61,473																			
**Previous knee infection**		4.86	[2.71-7.64]	<0.0001	<0.0001	5.47	[0.14-20.34]	0.38	0.63	9.71	[1.16-27.27]	0.01	0.06	1.90	[0.05-7.04]	0.96	0.98	5.00	[1.36-11.03]	0.01	0.03	5.10	[2.04-9.52]	<0.0001	<0.0001
DIC		53,355				61,464																			
**Avascular necrosis**		1.56	[0.97-2.33]	0.06	0.17	1.15	[0.03-4.23]	0.73	0.89	2.33	[0.28-6.51]	0.48	0.70	3.15	[1.26-5.93]	0.01	0.04	1.47	[0.48-3.02]	0.55	0.77	1.03	[0.38-2.00]	0.89	0.95
DIC		53,375				61,484																			
**Inflammatory arthropathy**		1.38	[1.15-1.65]	<0.0001	0.01	2.04	[0.91-3.66]	0.07	0.19	1.98	[0.93-3.45]	0.06	0.18	1.24	[0.71-1.91]	0.47	0.70	1.22	[0.79-1.74]	0.37	0.61	1.43	[1.09-1.83]	0.01	0.04
DIC		53,368				61,477																			
**Other indication**		1.36	[0.85-1.99]	0.19	0.40	0.82	[0.02-3.06]	0.54	0.75	1.45	[0.17-4.06]	0.90	0.96	0.57	[0.07-1.60]	0.31	0.56	1.18	[0.43-2.31]	0.86	0.94	1.84	[0.94-3.05]	0.06	0.18
DIC		53,377				61,492																			
**Surgical approach**	**Lateral parapatellar**	1.08	[0.79-1.42]	0.64	0.83	1.66	[0.44-3.69]	0.49	0.72	4.39	[2.16-7.45]	<0.0001	<0.0001	1.32	[0.60-2.34]	0.52	0.74	0.84	[0.38-1.48]	0.51	0.74	0.68	[0.37-1.09]	0.13	0.31
	**Midvastus**	0.82	[0.64-1.02]	0.08	0.23	0.93	[0.33-1.87]	0.71	0.88	0.76	[0.24-1.58]	0.42	0.66	0.45	[0.19-0.82]	0.02	0.08	0.88	[0.56-1.28]	0.48	0.70	0.86	[0.59-1.19]	0.36	0.61
	**Subvastus**	0.91	[0.67-1.19]	0.47	0.70	0.82	[0.17-2.01]	0.56	0.77	0.94	[0.19-2.28]	0.71	0.88	1.35	[0.66-2.28]	0.43	0.67	0.98	[0.52-1.59]	0.82	0.93	0.78	[0.47-1.18]	0.25	0.49
	**Other**	1.01	[0.77-1.28]	1.00	1.00	1.02	[0.32-2.17]	0.85	0.93	1.16	[0.37-2.41]	0.93	0.97	0.96	[0.47-1.63]	0.77	0.91	0.98	[0.57-1.51]	0.84	0.93	1.00	[0.66-1.43]	0.92	0.97
	**Medial parapatellar**	Ref				Ref				Ref				Ref				Ref				Ref			
DIC		53,380				61,477																			
**Procedure**	**TKR uncemented**	0.71	[0.60-0.84]	<0.0001	<0.0001	0.94	[0.45-1.63]	0.72	0.88	1.56	[0.89-2.43]	0.11	0.27	0.62	[0.37-0.92]	0.03	0.10	0.86	[0.61-1.15]	0.30	0.56	0.55	[0.41-0.71]	<0.0001	<0.0001
	**TKR other**	0.89	[0.66-1.16]	0.38	0.63	1.15	[0.23-2.82]	0.95	0.98	0.37	[0.01-1.38]	0.22	0.45	1.30	[0.61-2.25]	0.53	0.75	1.14	[0.63-1.81]	0.72	0.88	0.81	[0.51-1.19]	0.29	0.55
	**Patellofemoral**	0.33	[0.19-0.50]	<0.0001	<0.0001					0.35	[0.01-1.30]	0.21	0.43	0.49	[0.13-1.09]	0.12	0.29	0.15	[0.02-0.43]	0.01	0.04	0.38	[0.18-0.68]	<0.0001	0.02
	**Unicondylar**	0.54	[0.46-0.61]	<0.0001	<0.0001	0.53	[0.27-0.88]	0.02	0.09	1.04	[0.63-1.55]	0.97	0.98	0.42	[0.27-0.60]	<0.0001	<0.0001	0.46	[0.33-0.61]	<0.0001	<0.0001	0.56	[0.45-0.68]	<0.0001	<0.0001
	**TKR cemented**	Ref				Ref				Ref				Ref				Ref				Ref			
DIC		53,270				61,357																			
**Type of constraint**	**Unconstrained Mobile**	1.02	[0.89-1.17]	0.79	0.92	1.43	[0.82-2.24]	0.20	0.42	1.29	[0.74-2.00]	0.38	0.63	0.93	[0.65-1.27]	0.63	0.82	1.16	[0.89-1.47]	0.29	0.55	0.97	[0.79-1.17]	0.71	0.88
	**Posterior Stabilised Fixed**	1.38	[1.27-1.50]	<0.0001	<0.0001	1.41	[1.01-1.90]	0.04	0.14	1.34	[0.96-1.80]	0.09	0.23	1.41	[1.16-1.69]	<0.0001	0.01	1.32	[1.12-1.53]	<0.0001	0.01	1.40	[1.24-1.59]	<0.0001	<0.0001
	**Posterior Stabilised Mobile**	0.90	[0.67-1.18]	0.44	0.68	0.86	[0.17-2.14]	0.60	0.81	0.91	[0.18-2.24]	0.66	0.85	0.96	[0.43-1.71]	0.78	0.91	0.85	[0.44-1.42]	0.50	0.73	1.03	[0.67-1.48]	0.96	0.98
	**Constrained Condylar**	3.50	[2.52-4.65]	<0.0001	<0.0001	4.02	[1.07-9.02]	0.02	0.09	3.30	[0.66-8.11]	0.11	0.28	2.75	[1.10-5.20]	0.02	0.08	3.60	[1.90-5.87]	<0.0001	<0.0001	3.50	[1.99-5.46]	<0.0001	<0.0001
	**Fixed**	0.72	[0.56-0.92]	0.01	0.04	1.06	[0.43-2.03]	0.97	0.98	1.17	[0.46-2.25]	0.85	0.93	0.69	[0.35-1.14]	0.17	0.38	0.56	[0.30-0.90]	0.03	0.10	0.70	[0.45-1.01]	0.07	0.19
	**Mobile**	0.57	[0.48-0.68]	<0.0001	<0.0001	0.39	[0.12-0.83]	0.03	0.12	1.10	[0.59-1.80]	0.83	0.93	0.37	[0.20-0.59]	<0.0001	<0.0001	0.50	[0.34-0.70]	<0.0001	<0.0001	0.65	[0.50-0.81]	<0.0001	<0.0001
	**Undetermined**	1.11	[0.90-1.33]	0.34	0.59	1.54	[0.65-2.86]	0.34	0.59	1.64	[0.73-2.96]	0.23	0.46	1.02	[0.57-1.60]	0.95	0.98	1.21	[0.80-1.70]	0.37	0.62	0.98	[0.71-1.30]	0.85	0.93
	**Unconstrained Fixed**	Ref				Ref				Ref				Ref				Ref				Ref			
DIC		53,216				61,333																			
**General Anaesthesia**		1.11	[1.04-1.20]	<0.0001	0.02	1.05	[0.79-1.36]	0.79	0.92	1.27	[0.96-1.63]	0.09	0.24	1.19	[1.00-1.40]	0.05	0.16	1.12	[0.98-1.28]	0.09	0.24	1.13	[1.01-1.25]	0.03	0.11
DIC		53,372				61,473																			
**Nerve Block Anaesthesia**		1.02	[0.94-1.11]	0.60	0.81	1.23	[0.87-1.67]	0.24	0.48	1.00	[0.70-1.37]	0.94	0.98	1.03	[0.84-1.26]	0.78	0.91	1.02	[0.87-1.20]	0.80	0.92	1.00	[0.88-1.14]	0.97	0.98
DIC		53,378				61,485																			
**Epidural Anaesthesia**		0.89	[0.79-0.99]	0.04	0.14	0.55	[0.28-0.92]	0.03	0.12	1.06	[0.64-1.59]	0.90	0.96	0.82	[0.58-1.09]	0.18	0.39	1.03	[0.82-1.27]	0.83	0.93	0.99	[0.84-1.16]	0.88	0.95
DIC		53,374				61,484																			
**Spinal Anaesthesia**		0.90	[0.84-0.97]	<0.0001	0.02	1.06	[0.80-1.37]	0.75	0.90	0.77	[0.59-0.99]	0.04	0.14	0.84	[0.71-0.99]	0.04	0.14	0.89	[0.78-1.02]	0.09	0.24	0.88	[0.79-0.97]	0.01	0.06
DIC		53,371				61,466																			
**Thrombop·regimen**	**Not chemical**	0.88	[0.78-0.98]	0.02	0.09	1.00	[0.62-1.49]	0.92	0.97	0.96	[0.59-1.42]	0.75	0.90	1.02	[0.76-1.32]	0.95	0.98	0.96	[0.77-1.19]	0.70	0.88	0.93	[0.79-1.08]	0.33	0.59
	**Chemical**	Ref				Ref				Ref				Ref				Ref				Ref			
DIC		53,373				61,484																			
**Tibial bone graft**		1.95	[1.31-2.71]	<0.0001	<0.0001	2.92	[0.60-7.11]	0.16	0.37	0.96	[0.02-3.59]	0.63	0.82	1.50	[0.41-3.28]	0.61	0.81	3.22	[1.72-5.21]	<0.0001	<0.0001	1.35	[0.62-2.38]	0.47	0.70
DIC		53,368				61,476																			
**Femoral bone graft**		1.56	[1.00-2.25]	0.04	0.14	1.51	[0.18-4.25]	0.86	0.94					0.87	[0.18-2.11]	0.62	0.81	1.79	[0.81-3.15]	0.13	0.31	1.82	[0.87-3.11]	0.09	0.25
DIC		53,375				61,483																			
**Intra operative event**		1.19	[0.75-1.73]	0.48	0.70	2.40	[0.48-5.93]	0.28	0.54					1.14	[0.31-2.53]	1.00	1.00	1.46	[0.63-2.67]	0.39	0.63	0.98	[0.42-1.77]	0.82	0.93
DIC		53,378				61,486																			

RR: Rate ratio adjusted for age, sex, BMI and ASA grade; CI: 95% Confidence Interval; Adj. p-value: q-value or adjusted p-value. DIC: Deviance Information Criterion (RRs and 95%CI for ≤3mths to ≥24mths periods are estimated from the same piece-wise exponential multilevel model) TKR: Total knee replacement

Appendix Table 4: Incidence rate ratios of revision for PJI and 95% confidence intervals for health system characteristics

		Total				≤3mths				3–6mths				6–12mths				12–24mths				≥24mths			
		**RR**	**95%CI**	**P-value**	**Adj. p-value**	**RR**	**95%CI**	**P-value**	**Adj. p-value**	**RR**	**95%CI**	**P-value**	**Adj. p-value**	**RR**	**95%CI**	**P-value**	**Adj. p-value**	**RR**	**95%CI**	**P-value**	**Adj. p-value**	**RR**	**95%CI**	**P-value**	**Adj. p-value**
**Place of surgery**	**Wales**	0.88	[0.70-1.08]	0.21	0.43	1.22	[0.59-2.18]	0.67	0.85	0.77	[0.37-1.34]	0.34	0.59	0.66	[0.41-0.99]	0.05	0.16	0.80	[0.56-1.10]	0.17	0.38	1.00	[0.77-1.29]	0.98	0.99
	**England**	Ref				Ref				Ref				Ref				Ref				Ref			
DIC		53,378				61,487																			
**Funding**	**Private**	0.85	[0.75-0.95]	0.01	0.03	1.12	[0.71-1.64]	0.67	0.86	1.36	[0.91-1.92]	0.13	0.31	1.14	[0.87-1.46]	0.34	0.59	0.76	[0.59-0.96]	0.02	0.09	0.71	[0.59-0.85]	<0.0001	<0.0001
	**Unspecified**	0.84	[0.72-0.97]	0.02	0.09	1.04	[0.48-1.84]	0.96	0.98	0.81	[0.34-1.49]	0.46	0.70	1.00	[0.64-1.44]	0.92	0.97	1.03	[0.74-1.38]	0.90	0.96	0.90	[0.73-1.09]	0.29	0.55
	**NHS**	Ref				Ref				Ref				Ref				Ref				Ref			
DIC		53,371				61,482																			
**Grade operating surgeon**	**Other**	0.99	[0.90-1.08]	0.79	0.92	0.84	[0.55-1.19]	0.32	0.57	1.39	[0.99-1.88]	0.05	0.16	1.01	[0.80-1.25]	0.95	0.98	1.14	[0.95-1.34]	0.16	0.36	0.98	[0.85-1.12]	0.78	0.91
	**Consultant**	Ref				Ref				Ref				Ref				Ref				Ref			
DIC		53,378				61,489																			
**Consultant involved**	**None involved**	0.96	[0.86-1.07]	0.47	0.70	0.67	[0.38-1.07]	0.11	0.27	1.33	[0.86-1.92]	0.20	0.41	1.08	[0.81-1.39]	0.62	0.81	1.14	[0.92-1.39]	0.22	0.44	0.96	[0.81-1.12]	0.56	0.77
	**Assisting**	1.04	[0.90-1.19]	0.61	0.81	1.11	[0.61-1.76]	0.81	0.92	1.49	[0.88-2.30]	0.13	0.31	0.90	[0.61-1.26]	0.52	0.74	1.12	[0.85-1.44]	0.44	0.68	1.04	[0.83-1.27]	0.77	0.91
	**Operating**	Ref				Ref				Ref				Ref				Ref				Ref			
DIC		53,378				61,489																			
**Total volume Operating surgeon**	**>85**	0.95	[0.86-1.04]	0.26	0.51	1.24	[0.83-1.78]	0.31	0.57	0.91	[0.61-1.29]	0.54	0.75	0.90	[0.72-1.12]	0.34	0.59	0.74	[0.61-0.88]	<0.0001	0.01	0.86	[0.73-0.99]	0.04	0.14
	**]50-85]**	0.96	[0.87-1.05]	0.34	0.59	1.23	[0.83-1.75]	0.33	0.59	0.86	[0.58-1.22]	0.37	0.61	0.99	[0.79-1.22]	0.85	0.93	0.78	[0.65-0.93]	0.01	0.03	0.88	[0.76-1.00]	0.06	0.18
	**]25-50]**	1.09	[1.00-1.19]	0.05	0.16	1.25	[0.85-1.78]	0.28	0.54	1.18	[0.82-1.65]	0.41	0.65	0.81	[0.64-1.01]	0.06	0.19	1.00	[0.85-1.19]	0.99	1.00	1.14	[1.01-1.29]	0.04	0.14
	**≤25**	Ref				Ref				Ref				Ref				Ref				Ref			
DIC		53,373				61,469																			
**Tot volume Surgeon in charge**	**>110**	0.96	[0.87-1.06]	0.46	0.70	1.21	[0.82-1.74]	0.37	0.61	0.88	[0.60-1.25]	0.45	0.69	0.94	[0.75-1.18]	0.57	0.79	0.78	[0.64-0.93]	0.01	0.03	0.88	[0.75-1.02]	0.08	0.23
	**]70-110]**	0.99	[0.90-1.09]	0.84	0.93	1.13	[0.76-1.63]	0.58	0.79	0.98	[0.67-1.39]	0.84	0.93	0.99	[0.79-1.23]	0.88	0.95	0.89	[0.74-1.05]	0.17	0.38	0.86	[0.74-0.99]	0.03	0.12
	**]38-70]**	1.02	[0.94-1.11]	0.64	0.83	1.29	[0.89-1.82]	0.19	0.41	1.04	[0.72-1.46]	0.88	0.95	0.90	[0.72-1.12]	0.34	0.59	0.86	[0.72-1.01]	0.07	0.19	1.04	[0.92-1.18]	0.52	0.74
	**≤38**	Ref				Ref				Ref				Ref				Ref				Ref			
DIC		53,379				61,482																			
**Total volume Hospital**	**>440**	1.25	[1.10-1.42]	<0.0001	0.01	2.09	[1.31-3.18]	<0.0001	0.01	0.95	[0.63-1.37]	0.71	0.88	1.03	[0.79-1.32]	0.87	0.94	1.00	[0.80-1.23]	0.94	0.98	1.00	[0.84-1.19]	0.97	0.98
	**]285-440]**	1.24	[1.11-1.37]	<0.0001	<0.0001	1.28	[0.82-1.90]	0.30	0.56	0.98	[0.66-1.40]	0.84	0.93	1.01	[0.79-1.27]	1.00	1.00	1.16	[0.95-1.40]	0.15	0.35	1.11	[0.96-1.29]	0.17	0.38
	**]150-285]**	1.15	[1.04-1.26]	0.01	0.03	1.21	[0.79-1.77]	0.42	0.66	0.98	[0.67-1.39]	0.83	0.93	0.96	[0.76-1.20]	0.68	0.86	1.14	[0.94-1.36]	0.19	0.40	1.10	[0.96-1.25]	0.20	0.41
	**≤150**	Ref				Ref				Ref				Ref				Ref				Ref			
DIC		53,353				61,474																			

RR: Rate ratio adjusted for age, sex, BMI and ASA grade; CI: 95% Confidence Interval; Adj. p-value: q-value or adjusted p-value. DIC: Deviance Information Criterion (RRs and 95%CI for ≤3mths to ≥24mths periods are estimated from the same piece-wise exponential multilevel model)

STROBE Statement—checklist of items that should be included in reports of observational studies

	Item No	Recommendation
**Title and abstract**	1	(*a*) Indicate the study's design with a commonly used term in the title or the abstract **Page1**
		(*b*) Provide in the abstract an informative and balanced summary of what was done and what was found **Page4**
**Introduction**		
Background/rationale	2	Explain the scientific background and rationale for the investigation being reported **Page5**
Objectives	3	State specific objectives to including any prespecified hypotheses **Page5**
**Methods**		
Study design	4	Present key elements of study design early in the paper **Page6**
Setting	5	Describe the setting to locations to and relevant dates to including periods of recruitment to exposure to follow-up to and data collection **Page6**
Participants	6	(*a*) *Cohort study*—Give the eligibility criteria to and the sources and methods of selection of participants. Describe methods of follow-up **Page6**<br>*Case-control study*—Give the eligibility criteria to and the sources and methods of case ascertainment and control selection. Give the rationale for the choice of cases and controls<br>*Cross-sectional study*—Give the eligibility criteria to and the sources and methods of selection of participants
		(*b*) *Cohort study*—For matched studies to give matching criteria and number of exposed and unexposed<br>*Case-control study*—For matched studies to give matching criteria and the number of controls per case
Variables	7	Clearly define all outcomes to exposures to predictors to potential confounders to and effect modifiers. Give diagnostic criteria to if applicable **Page6**
Data sources/ measurement	8*	For each variable of interest to give sources of data and details of methods of assessment (measurement). Describe comparability of assessment methods if there is more than one group **Page6**
Bias	9	Describe any efforts to address potential sources of bias **Page7**
Study size	10	Explain how the study size was arrived at **Page6 Figure1**
Quantitative variables	11	Explain how quantitative variables were handled in the analyses. If applicable to describe which groupings were chosen and why **Page6**
Statistical methods	12	(*a*) Describe all statistical methods to including those used to control for confounding **Page7**
		(*b*) Describe any methods used to examine subgroups and interactions **Page7**
		(*c*) Explain how missing data were addressed **Page7**
		(*d*) *Cohort study*—If applicable to explain how loss to follow-up was addressed **Page7**<br>*Case-control study*—If applicable to explain how matching of cases and controls was addressed<br>*Cross-sectional study*—If applicable to describe analytical methods taking account of sampling strategy
		(*e*) Describe any sensitivity analyses **Page7**

Continued on next page

**Results**		
Participants	13*	(a) Report numbers of individuals at each stage of study—eg numbers potentially eligible to examined for eligibility to confirmed eligible to included in the study to completing follow-up to and analysed **Figure1 Page9**
		(b) Give reasons for non-participation at each stage **Figure1**
		(c) Consider use of a flow diagram **Figure1**
Descriptive data	14*	(a) Give characteristics of study participants (eg demographic to clinical to social) and information on exposures and potential confounders **Figure1+tables in doc**
		(b) Indicate number of participants with missing data for each variable of interest **Tables in doc+Tables in appendix**
		(c) *Cohort study*—Summarise follow-up time (eg to average and total amount) **Tables in doc+Tables in appendix**
Outcome data	15*	*Cohort study*—Report numbers of outcome events or summary measures over time **Tables in doc+Tables in appendix**
		Case-control study—Report numbers in each exposure category to or summary measures of exposure
		Cross-sectional study—Report numbers of outcome events or summary measures
Main results	16	(*a*) Give unadjusted estimates and to if applicable to confounder-adjusted estimates and their precision (eg to 95% confidence interval). Make clear which confounders were adjusted for and why they were included **Tables in doc+Tables in appendix+Figures 2-3 & Figures in appendix**
		(*b*) Report category boundaries when continuous variables were categorized **Tables in doc+Tables in appendix+Figures 2-3 & Figures in appendix**
		(*c*) If relevant to consider translating estimates of relative risk into absolute risk for a meaningful time period
Other analyses	17	Report other analyses done—eg analyses of subgroups and interactions to and sensitivity analyses
**Discussion**		
Key results	18	Summarise key results with reference to study objectives **Page12**
Limitations	19	Discuss limitations of the study to taking into account sources of potential bias or imprecision. Discuss both direction and magnitude of any potential bias **Page13**
Interpretation	20	Give a cautious overall interpretation of results considering objectives to limitations to multiplicity of analyses to results from similar studies to and other relevant evidence **Pages12-13**
Generalisability	21	Discuss the generalisability (external validity) of the study results evidence **Pages13-14**
**Other information**		
Funding	22	Give the source of funding and the role of the funders for the present study and to if applicable to for the original study on which the present article is based **Page8+Page15**

*Give information separately for cases and controls in case-control studies and to if applicable to for exposed and unexposed groups in cohort and cross-sectional studies.

Note: An Explanation and Elaboration article discusses each checklist item and gives methodological background and published examples of transparent reporting. The STROBE checklist is best used in conjunction with this article (freely available on the Web sites of PLoS Medicine at http://www.plosmedicine.org/ to Annals of Internal Medicine at http://www.annals.org/ to and Epidemiology at http://www.epidem.com/). Information on the STROBE Initiative is available at www.strobe-statement.org.

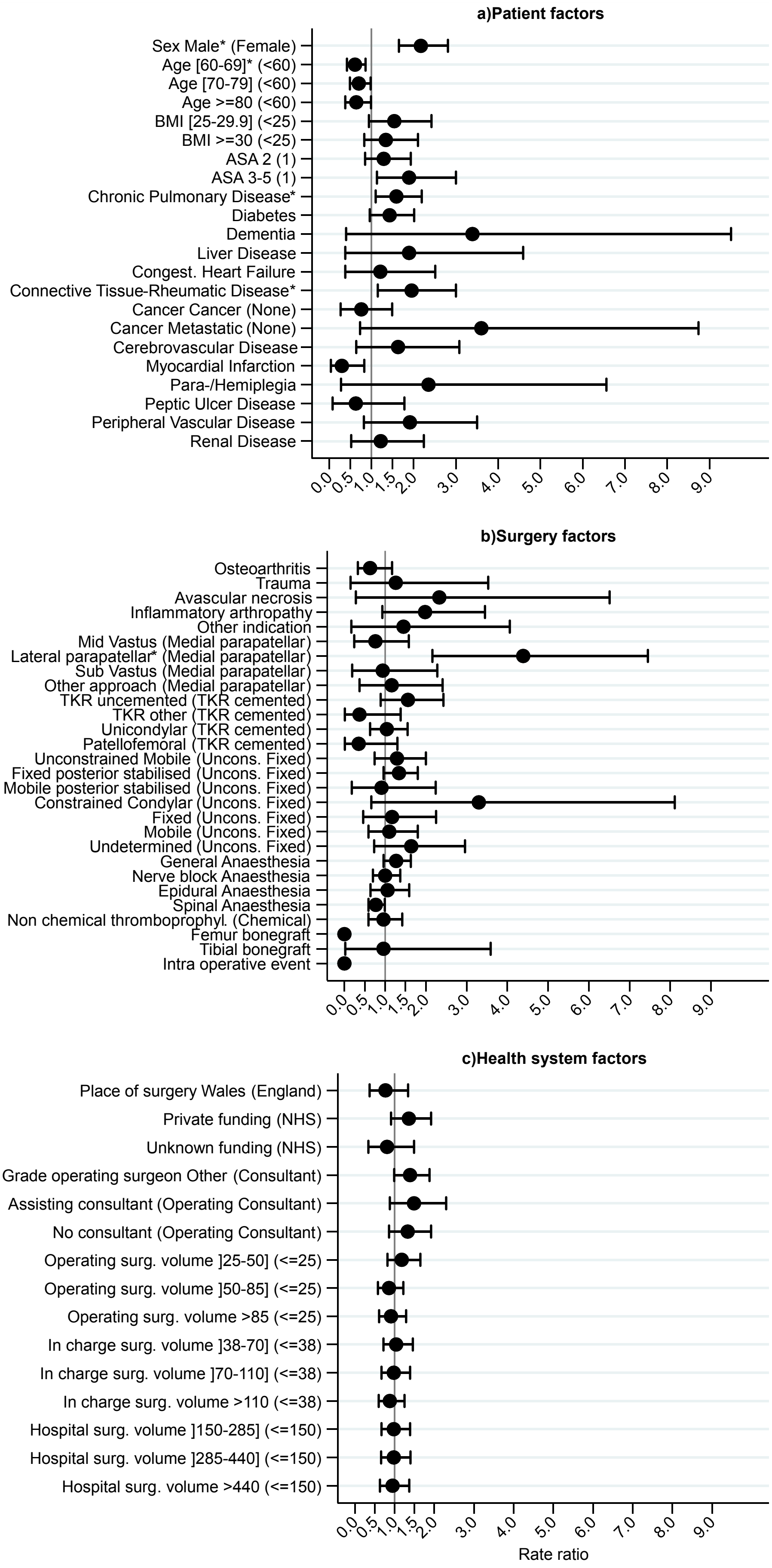

Appendix Figure 1: Risk factors of revision for prosthetic joint infection for the 3-6 postoperative months (Reference category in bracket)

*Adjusted p-value<0.05, detailed in Appendix tables 2-4 alongside the rate ratios and confidence intervals. BMI: Body Mass Index, ASA: American Society of Anaesthesiologists, TKR: Total knee replacement

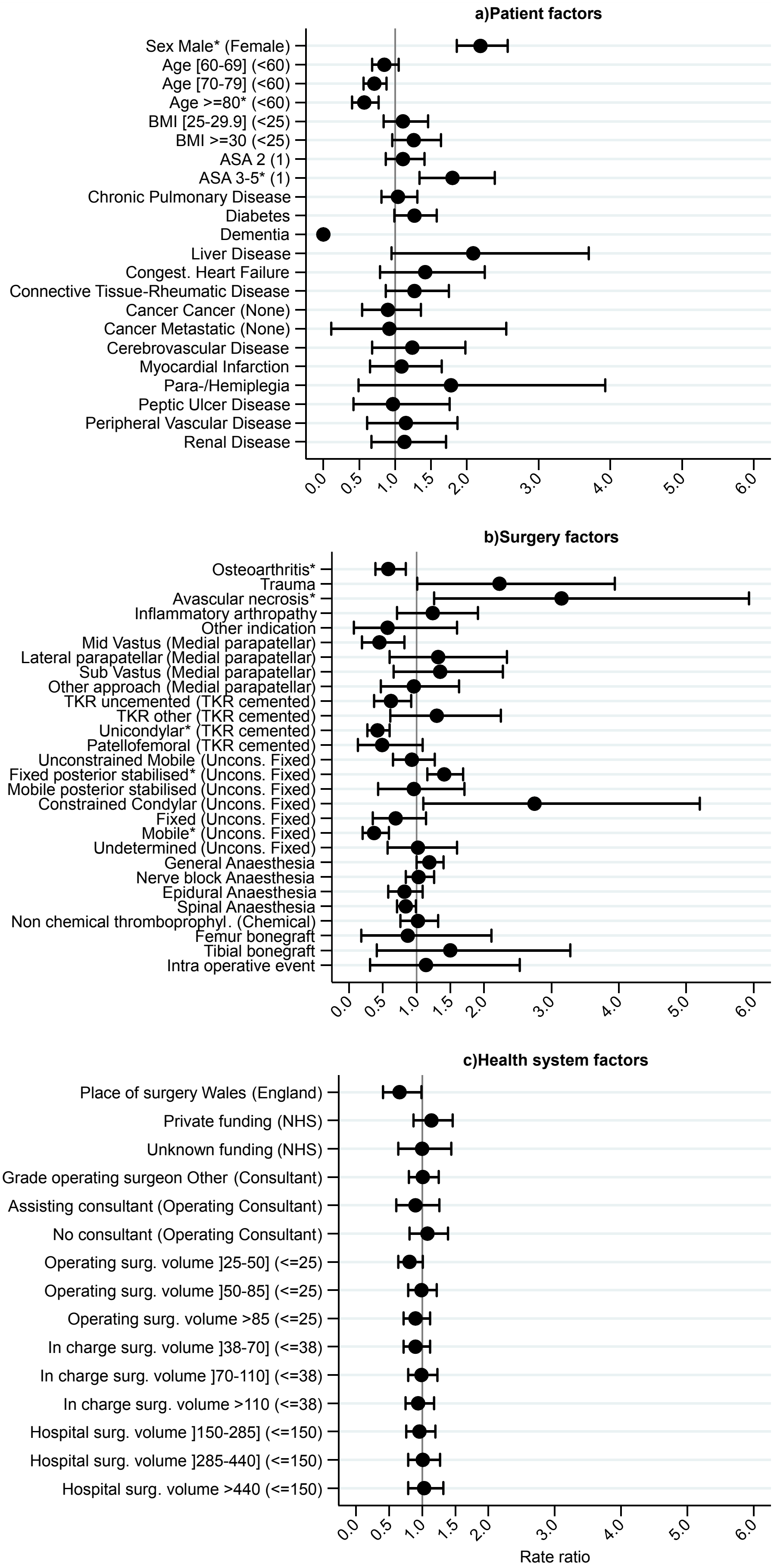

Appendix Figure 2: Risk factors of revision for prosthetic joint infection for the 6-12 postoperative months (Reference category in bracket)

*Adjusted p-value<0.05, detailed in Appendix tables 2-4 alongside the rate ratios and confidence intervals. BMI: Body Mass Index, ASA: American Society of Anaesthesiologists, TKR: Total knee replacement

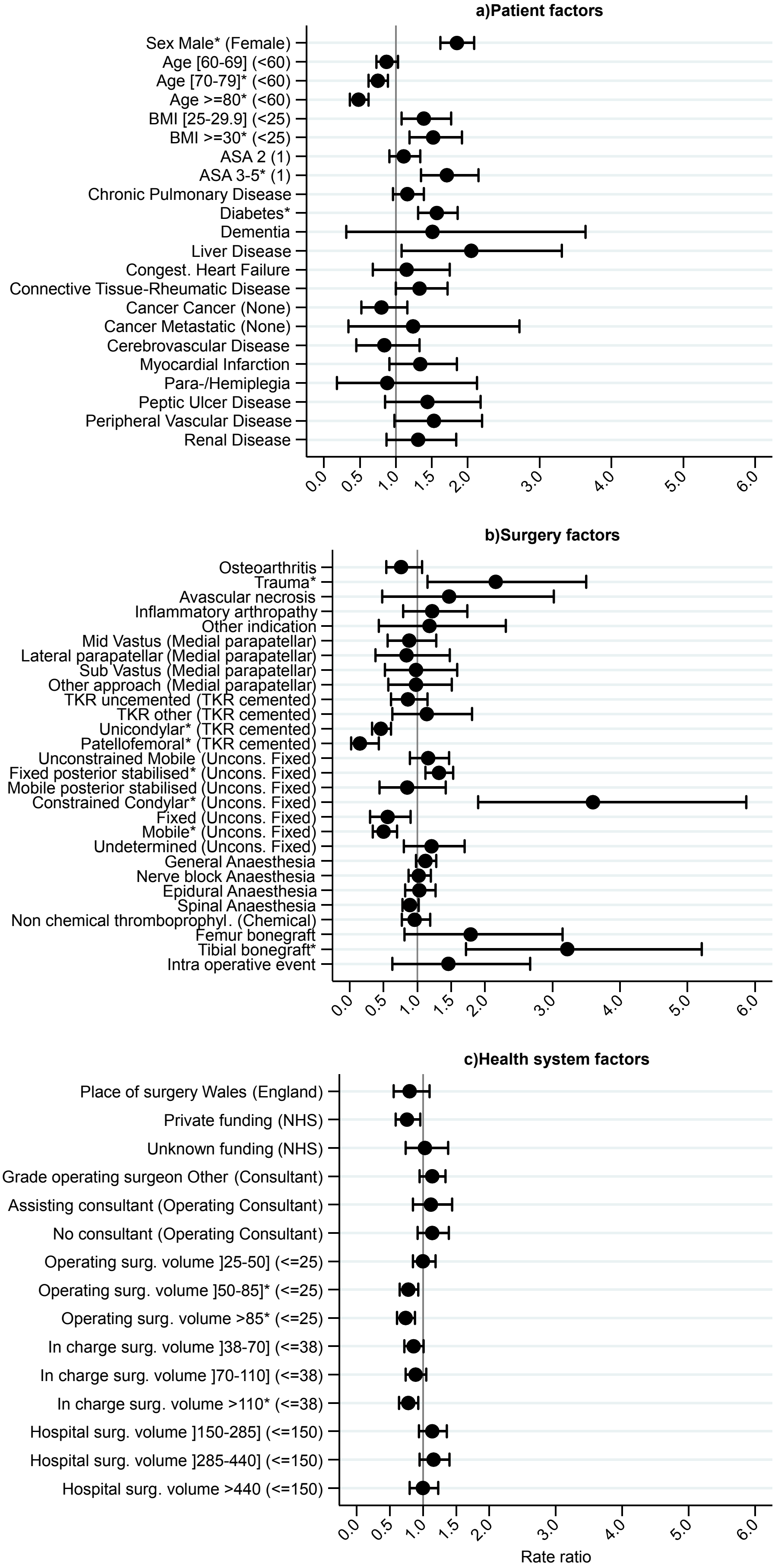

Appendix Figure 3: Risk factors of revision for prosthetic joint infection for the 12-24postoperative months (Reference category in bracket)

*Adjusted p-value<0.05, detailed in Appendix tables 2-4 alongside the rate ratios and confidence intervals. BMI: Body Mass Index, ASA: American Society of Anaesthesiologists, TKR: Total knee replacement

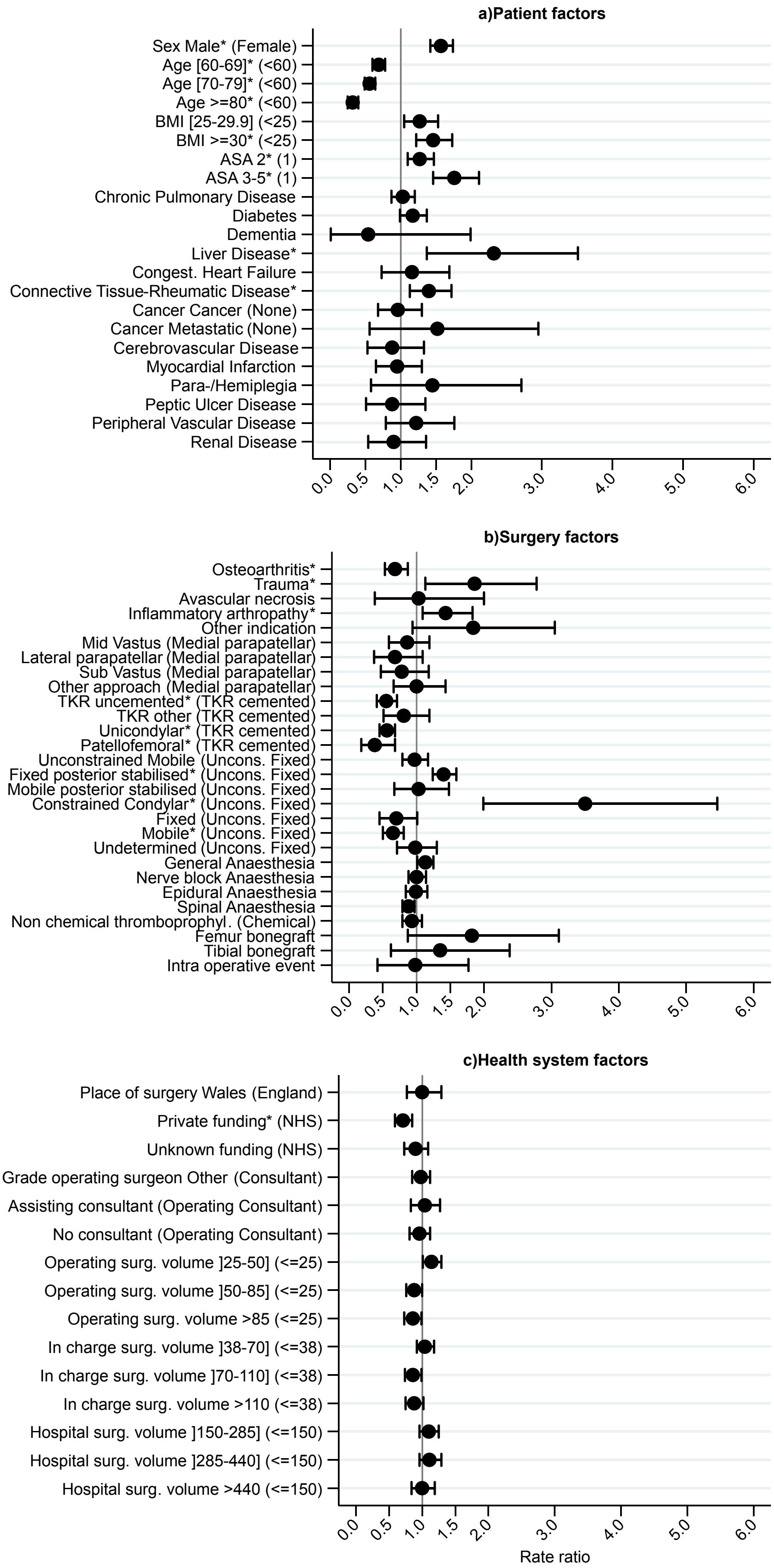

Appendix Figure 4: Risk factors of revision for prosthetic joint infection for the ≥24postoperative months (Reference category in bracket)

*Adjusted p-value<0.05, detailed in Appendix tables 2-4 alongside the rate ratios and confidence intervals. BMI: Body Mass Index, ASA: American Society of Anaesthesiologists, TKR: Total knee replacement
